# Research priorities for back pain: Results from an adapted James Lind Alliance priority setting partnership

**DOI:** 10.1371/journal.pone.0353164

**Published:** 2026-07-13

**Authors:** Rikke Munk Killingmo, Ingrid Fjeldheim Bånerud, Astrid Torgersen Lunestad, Anne Marie Selboskar Selven, Eli Steen, Ann Iren Eikrem, Inger Ljøstad, Thale Skogstad, Kjersti Storheim, Amy Martinsen

**Affiliations:** 1 Department of Research and Innovation, Division of Clinical Neuroscience, Oslo University Hospital, Oslo, Norway; 2 Norwegian Spine and Back Pain Association, Oslo, Norway; 3 Department of Rehabilitation Science and Health Technology, Oslo Metropolitan University, Oslo, Norway; 4 Norwegian Chiropractors`Association, Oslo, Norway; University of Toronto, CANADA

## Abstract

**Background:**

Knowledge gaps remain in the field of back pain research, with few systematic efforts undertaken to identify research priorities that reflect the preferences of individuals affected by the condition. The objective of this study was to identify the top 10 research priorities for back pain, as defined by individuals with lived experience of back pain and healthcare professionals.

**Method:**

A James Lind Alliance Priority Setting Partnership approach was conducted in Norway. This involved (1) establishing a Steering Committee; (2) defining a scope; (3) gathering stakeholders’ evidence uncertainties about back pain using focus group interviews; (4) summarising evidence uncertainties and verifying uncertainties by checking existing evidence; (5) shortlisting evidence uncertainties using an online survey; and (6) determining the top 10 research priorities through a priority-setting workshop.

**Results:**

In total, 230 questions were generated through three focus group interviews involving 12 individuals with lived experience of back pain and seven healthcare professionals. Generated questions were summarised into 37 core questions. None of the core questions had been sufficiently addressed by existing evidence (prioritising high-quality evidence, preferably systematic reviews). The shortlisting survey was completed by 284 respondents (263 individuals with lived experience of back pain (93%), and 40 healthcare professionals (14%), with some respondents identifying as both). The top 25 core questions were brought forward to the priority-setting workshop involving five healthcare professionals and four individuals with lived experience of back pain. The top research priority was “How can implementation of evidence-based treatment recommendations for back pain be ensured in the Norwegian healthcare system?”.

**Conclusion:**

Adopting the JLA approach, this study has identified the top 10 research priorities for back pain from the perspectives of both individuals with lived experience of back pain and healthcare professionals. These findings should be considered a guide to research areas that are meaningful to end-users.

## Introduction

Back pain is common across the lifespan [1,2], and characterised by substantial heterogeneity in symptom presentation and clinical course. Symptoms range from mild to severe and disabling pain, and duration may vary from short episodes to long-lasting conditions [3]. The median lifetime prevalence of low back pain has been estimated at 42% globally [4]. While mild back pain tends to decline after midlife, severe and disabling back pain becomes more common with increasing age [5–7]. Back pain is the leading cause of disability globally [8,9], and alongside a high individual disease burden, back pain also creates a major socio-economic burden related to healthcare utilization and productivity loss [10–12]. With an aging population and an increasing number of older individuals with back pain, this burden is likely to increase in the years to come [11,12].

To reduce the burden of back pain and improve healthcare-related decision-making, more research is needed [13]. Traditionally, research agendas and priorities have been determined by researchers, research institutions or funding organizations [14]. However, evidence suggest that this traditional approach does not always align with the needs and preferences of end-users [15]. Back pain is a complex condition, influenced by multiple factors and characterised by substantial heterogeneity in symptoms and clinical course [2]. Therefore, research priorities established without end-user involvement risk overlooking issues most relevant to those living with the condition and their healthcare providers. Consequently, there is now a strong recommendation for researchers to actively involve non-research stakeholders in setting the scientific research agenda [14,16]. To address the lack of end-user involvement in research, the James Lind Alliance (JLA) [17] was established to develop a structured framework for Priority Setting Partnerships (PSPs) which provides a platform for collaboration between end-users and researchers in shaping future research agendas [17]. Within the PSP process, different methods are being used to gather questions from end-users and identify research priorities aimed at addressing unresolved uncertainties [17]. Research priorities can vary across social groups and geographical regions, both within and between countries, underscoring the importance of national initiatives to identify context-specific uncertainties [18].

While a few studies have explored research priorities in specific back-related diagnoses, such as spinal cord injury and scoliosis [19–22], no study has examined research priorities related to the broad population of individuals with back pain using the JLA PSP approach. Therefore, the aim of this study was to identify the top 10 research priorities for back pain, as defined by individuals with lived experience of back pain and healthcare professionals.

## Materials and methods

**Fig 1:**
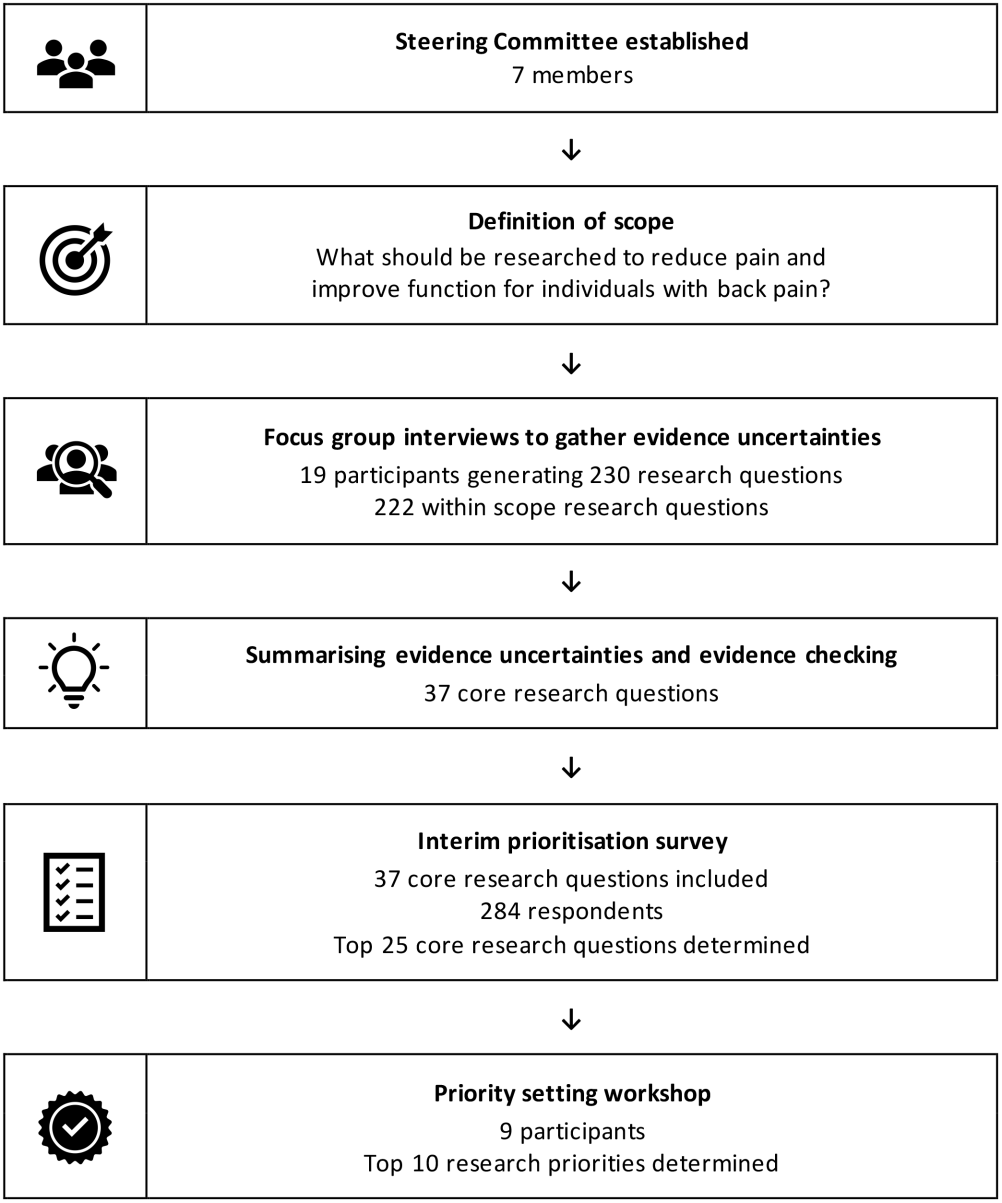
Flow chart of the Priority Setting Partnership.

This study was initiated by the Norwegian Spine and Back Pain Association as part of a collaborative project to enhance patient involvement in musculoskeletal research at Oslo University Hospital [23]. It was conducted as a PSP inspired by the JLA methodology [17] as outlined in Fig 1, from August 2024 to January 2025. The PSP has been reported following the REporting guideline for PRIority SEtting of health research (REPRISE) [24].

### Ethics

In accordance with the JLA Guidebook [17], ethical approval was not required for this study, as it involved research planning with stakeholder consultations rather than research conductance and participation [17]. Consultation with the Data Protection Officer at Oslo University Hospital (Oslo, Norway) confirmed that ethical approval and participant consent were unnecessary, as no identifiable data were collected and the material fell outside the scope of research data requiring ethical review in Norway. Nevertheless, the study was conducted in accordance with the Declaration of Helsinki. At each stage, participants were fully informed about the nature of their involvement as stakeholders and how their data would be used. They were assured that all collected data would be anonymised. All data were securely stored on the protected IT-platform at Oslo University Hospital.

### Establishing a steering committee

A Steering Committee, chaired by one of the project leaders (RMK), was established. Members were selected and invited through the Norwegian Spine and Back Pain Association, ensuring a diverse and representative group with varied experience and expertise on back pain and related research. The committee included seven members: three patient representatives with lived experience of back pain, two healthcare professionals (one medical doctor and one chiropractor), and two project leaders – one with clinical and research expertise (RMK) and one experienced patient representative with prior experience conducting a PSP using the JLA methodology (ATL).

### Defining scope

At the initial meeting of the Steering Committee, the scope of the PSP was formally confirmed as: “What research is needed to find better ways to reduce pain and improve function for individuals with back pain? By back pain, we refer to pain located in the region from the top of the scapula to the first sacral vertebra, to the extent that the individual has required or currently requires treatment, or experiences disability.”

### Gathering evidence uncertainties

Three focus group interviews were conducted to invite individuals with lived experience of back pain and healthcare professionals to suggest evidence of uncertainties within the scope of the PSP. Participants were eligible if they either had lived experience of back pain or were healthcare professionals proving care for patients with back pain. Participants were recruited by the Norwegian Spine and Back Pain Association through its networks, in consultation with the project leaders (RMK and ATL) at Oslo University Hospital. Recruitment aimed to ensure diversity. For individuals with lived experience, variation in age, sex, place of residence, native language, and diagnosis (non-specific and specific, e.g., scoliosis) was sought. For healthcare professionals, recruitment aimed to include different professions, as well as representatives from primary and specialist healthcare settings, with variation in age and sex.

A diverse range of participants was included in each focus group to ensure a balanced representation of all stakeholders and reduce the risk of bias in question weighting. Focus group one (conducted face-to-face) and two (conducted online via the Zoom platform to facilitate recruitment from rural areas of Norway) consisted of individuals with lived experience of back pain. Focus group three (conducted face-to-face) consisted of healthcare professionals caring for patients with back pain. All focus groups were moderated by the two project leaders (RMK and ATL). All participants were financially remunerated for their participation.

### Summarising evidence uncertainties

All suggested research questions were classified as either “within scope” or “out of scope” by one of the project leaders (RMK). Within scope, research questions with common themes were grouped together to form a single core research question [17]. The core research questions were designed to be clear and easily understandable, addressing a broad topic while accurately capturing the content provided by participants, rather than the specific research question. All core research questions were reviewed and agreed upon by consensus of the Steering Committee. A preference was given to keeping the core research questions broad and inclusive, rather than formulating multiple specific research questions. All core research questions underwent an evidence review to determine if they had been sufficiently addressed by existing literature. PubMed was used as the primary search database, with priority given to high-quality evidence, preferably systematic reviews. The search strategy was guided by the wording of each individual question, with search terms derived from the key concepts of the question and supplemented by closely related or synonymous terms to ensure comprehensive coverage. If it was unclear whether a core research question had been sufficiently addressed in the literature, the question and relevant literature were review by the Steering Committee for consensus. A question was retained unless strong evidence indicated that it had already been thoroughly addressed in previous research. All unanswered core research questions were included in the interim prioritisation survey.

### Interim prioritisation

An online survey (Nettskjema.no) was used to rank the core research questions and generate a shortlist of the most important ones to be discussed at the final priority-setting workshop. The core research questions were organized into five theme categories (1: Causation, risk factors, prevention and prognosis; 2: Examination and diagnosis; 3: Treatment; 4: Recognition and attitude; and 5: Public health measures and structure of the healthcare system) and presented in a random order unique to each respondent. Respondents were asked to rate each core research question and each theme category based on its importance using a scale from 1 to 5 (1: Unimportant; 2: Neutral; 3: Slightly important; 4: Quite important; and 5: Very important). Additionally, routine demographic data were collected. The survey remained open for four weeks, during which it was promoted to individuals with lived experience of back pain, as well as healthcare professionals involved in the care of patients with back pain. Promoting efforts included outreach through the Norwegian Spine and Back Pain Association, social media platforms, and the network of the Steering Committee. Following this, the raw data were analysed to identify the core research questions with the highest overall score of importance. To account for potential differences in the rating of the theme categories, each core question was additionally weighted by the overall rating of its respective theme category. Both the top 20 core research questions, ranked without adjustment for theme category ratings, and the top 20 core research questions, ranked with adjustments for theme category ratings, were forward to the final priority-setting workshop.

### Priority setting-workshop

The priority-setting workshop was conducted in person, bringing together individuals with lived experience of back pain and healthcare professionals involved in the care of patients with back pain, to determine the final top 10 priorities. A diverse range of participants was included to ensure balanced representation of all stakeholders and reduce the risk of bias in question weighting. Participants were provided with the shortlisted core research questions prior to the workshop. During the workshop, participants were divided into two groups, each with a balanced representation of all stakeholders. The workshop consisted of five phases, moderated by the two project leaders (RMK and ATL):

1. *Small group discussions:* Group members were given printed cards containing the core research questions (with the core research question on the front and the ranking score from the interim prioritisation survey on the back). They were asked to list the three core research questions they considered most important and the three they felt were least important.
2. *First round of small group ranking:* In the same groups, group members were asked to organize the core research questions into rough categories: those considered most important, those considered least important, and those deemed uncertain. Subsequently, group members prioritised all core research questions by moving the cards into ranked order.
3. *Plenary review:* The ranking agreed upon by each group were entered into a spreadsheet and assigned a numerical value (highest rank = 1, second highest = 2, etc.). The group rankings were then presented to all participants in plenary, with an opportunity for discussion.
4. *Second round of small group ranking:* In the same groups, group members were asked to discuss and revise the ranked list based on the plenary discussion. Cards were rearranged to reflect the new rank order.
5. *Final plenary review:* As in phase three, the small group rankings were entered into a spreadsheet and presented to all participants in plenary. The final ranking was discussed and agreed upon by all participants.

All discussions were chaired by the two project leaders (RMK and ATL) to ensure that no single group or individual dominated the decision-making process. The goal was to reach an agreement by consensus, with decisions made by majority vote if consensus could not be achieved. All participants were financially remunerated for their participation.

### Deviations from the JLA Guidebook

This study deviated from the JLA Guidebook [17] in four ways. First, although a protocol outlining the aim, scope, and method was developed, it was not registered or published on the JLA website, as the study was conducted independently rather than as a formally registered JLA PSP. Second, no formal JLA adviser was involved due to independence and lack of funding for this service. Third, carers were not included as stakeholder group, as back pain is typically managed in primary care and largely self-managed, with informal caregiving not being a central component of routine management in most cases [2]. Consequently, the perspectives of individuals with lived experience of back pain and healthcare professionals were prioritised. Fourth, uncertainties were identified through focus groups. While JLA PSPs typically use online surveys, focus groups and purposive recruitment were used to ensure participant diversity and enable in-depth discussion and collective reflection, revealing uncertainties that might not emerge through surveys. Thus, the use of focus groups rather than surveys constituted a deliberate adaptation of the traditional JLA PSP approach.

## Results

### Gathering evidence uncertainties and summarising evidence uncertainties

The three focus groups consisted of 12 individuals with lived experience of back pain (median age 53 (range 22–77) years; 8 (67%) women; 10 (83%) native Norwegian speakers) and seven healthcare professionals (physiotherapist, chiropractor, medical doctor, orthopaedic surgeon, neurologist, and physiologist) caring for patients with back pain. In total, 230 research questions were suggested (175 from individuals with lived experience of back pain and 55 from healthcare professionals). After initial review, 8 suggestions were deemed out of scope. The remaining 222 research questions were consolidated into 37 core research questions through consensus by the Steering Committee. None of the core research questions had been sufficiently addressed by existing literature.

### Interim prioritisation

The online survey received 284 responses, of which 263 (93%) identified as individuals with lived experience of back pain, and 40 (14%) as healthcare professionals, with some respondents holding dual roles. Table 1 summarises demographics of the respondents. The top 25 core research questions (including the top 20 ranked without adjustment for theme category ratings and the top 20 ranked with adjustments for theme category ratings) are presented in Table 2.

**Table 1:** Demographic of respondents to the interim prioritisation survey.

	All respondents (n = 284)	Patients (n = 263)	Healthcare professionals (n = 40)	Missing, n (%)
Female, n (%)	237 (84)	224 (85)	22 (55)	0 (0)
Age in years, median (IQR)	53 (44-63)	54 (44-63)	45 (37-50)	7 (3)
Pain duration, n (%)				0 (0)
0–3 months	–	12 (5)	–	
> 3–12 months	–	4 (1)	–	
> 1–3 years	–	16 (6)	–	
> 3–5 years	–	20 (8)	–	
> 5–10 years	–	32 (12)	–	
> 10 years	–	179 (68)	–	
Type of healthcare professional, n (%)				0 (0)
Chiropractor	–	–	3 (8)	
General practitioner	–	–	2 (5)	
Physiotherapist	–	–	24 (60)	
Naprapath	–	–	1 (2)	
Osteopath	–	–	10 (25)	
Competence regarding back pain, n (%)				0 (0)
Very high	–	–	17 (43)	
High	–	–	13 (33)	
Moderate	–	–	9 (22)	
Low	–	–	1 (2)	
Very low	–	–	0 (0)	

All values are presented by number (valid percentage of total) or median (IQR). Cells marked with a dash (-) indicate that the variable was not measured.

**Table 2:** The top 25 ranked core research questions from the interim prioritisation survey.

	Ranking without adjustment for theme category rating			Ranking with adjustment for theme category rating
	**All respondents**	**Patients**	**Healthcare professionals**	**All respondents**
How can we prevent back pain?	1	Joint 1	2	5
Which type of treatment measures for back pain work best for whom?	2	Joint 1	Joint 5	1
What are the long-term consequences of back pain?	3	3	10	8
What types of physical activity and exercise are appropriate for individuals with back pain?	4	4	Joint 13	2
What guidance and treatment do individuals with back pain receive within the Norwegian healthcare system?	Joint 5	5	Joint 7	3
How can the Norwegian healthcare system be organized to improve the diagnosis and treatment of individuals with back pain?	Joint 5	6	Joint 7	15
How can we increase knowledge among individuals with back pain, so they are better able to make more informed decisions in their own lives?	7	7	1	17
Would expedite diagnosis and treatment improve treatment outcomes and overall results for individuals with back pain?	8	8	Joint 11	18
Which factors increase the risk of back pain?	9	9	3	10
What should be the content of a patient-directed tool for self-management of back pain?	Joint 10	Joint 10	Joint 13	4
How can implementation of evidence-based treatment recommendations for back pain be ensured in the Norwegian healthcare system?	Joint 10	Joint 12	Joint 5	21
How should a multidisciplinary care pathway for individuals with back pain be organized, and what is the effect of such an initiative?	12	Joint 10	25	6
How can evidence-based treatment recommendations for back pain be most effectively communicated by healthcare professionals to individuals with back pain?	13	14	4	7
How can the recognition of back pain be increased within the Norwegian healthcare system?	Joint 14	Joint 12	Joint 34	28
How can consistency in the diagnosis and treatment of individuals with back pain be improved within the Norwegian healthcare system?	Joint 14	17	18	24
Does timing of treatment affect its effectiveness? What is the optimal “treatment window” for individuals with back pain?	16	16	Joint 19	9
What should be included in a clinician-directed support tool for diagnosing back pain?	Joint 17	Joint 19	Joint 11	13
Are back pain complaints trivialized within the Norwegian healthcare system, and what are the potential consequences of this?	Joint 17	15	Joint 34	30
What is the cause of “nonspecific” back pain?	Joint 19	Joint 19	Joint 19	14
How can knowledge about back pain be increased within school health services and ensure better accommodations for young individuals experiencing back pain?	Joint 19	18	Joint 21	26
What should be the content of a clinician-directed treatment tool for back pain?	22	24	Joint 16	11
What is the effect of treatment tools for back pain?	26	26	24	12
What should be included in a patient-directed support tool for diagnosing back pain?	24	Joint 22	Joint 21	16
Which factors increase the risk of children and adolescents with scoliosis having to undergo spinal surgery?	25	25	Joint 16	19
What is the effectiveness of a diagnostic tool for back pain?	27	27	Joint 26	20

Ranking, adjusted for theme category ratings, was calculated by multiplying the overall rating of each core question by the overall rating of its respective theme category. The overall rating for the theme categories were as follow: Theme category 1 (Causation, risk factors, prevention and prognosis) = 4.61; Theme category 2 (Examination and diagnosis) = 4.64; Theme category 3 (Treatment) = 4.81; Theme category 4 (Recognition and attitude) = 4.18); and Theme category 5 (Public health measures and structure of the healthcare system) = 4.37.

### Priority-setting workshop

The priority-setting workshop consisted of nine participants (five healthcare professionals and four individuals with lived experience of back pain) and two moderators (RMK and ATL). The healthcare professionals included two physiotherapists, one chiropractor, one osteopath and one physiologist. The moderators facilitated the discussions, offering guidance and clarity without influencing the outcomes. The 25 core research questions were ranked, and the final top 10 research priorities for back pain were agreed upon, as shown in Table 3. All decisions were made by consensus, with no majority votes required. The discussions were conducted in a respectful and collaborative atmosphere, with participants actively engaging with and remaining receptive to differing perspectives. The two project leaders did not observe any dominance by individual participants or groups during the process.

**Table 3:** The final top 10 unanswered research questions for back pain.

1	How can implementation of evidence-based treatment recommendations for back pain be ensured in the Norwegian healthcare system?
2	Would expedite diagnosis and treatment improve treatment outcomes and overall results for individuals with back pain?
3	How can the recognition of back pain be increased within the Norwegian healthcare system?
4	How can the Norwegian healthcare system be organized to improve the diagnosis and treatment of individuals with back pain?
5	Which type of treatment measures for back pain work best for whom?
6	How can we increase knowledge among individuals with back pain, so they are better able to make more informed decisions in their own lives?
7	How can consistency in the diagnosis and treatment of individuals with back pain be improved within the Norwegian healthcare system?
8	How can knowledge about back pain be increased within school health services and ensure better accommodations for young individuals experiencing back pain?
9	Which factors increase the risk of children and adolescents with scoliosis having to undergo spinal surgery?
10	What should be included in a clinician-directed support tool for diagnosing back pain?

## Discussion

This PSP has identified the top 10 research priorities for back pain by combining the perspectives of individuals with lived experience of back pain and healthcare professionals in Norway. These priorities address research uncertainties across multiple areas of research interest, including knowledge implementation (priorities 1, 6 and 8), healthcare system organization (priorities 2 and 4) and consistency (priority 7), recognition (priority 3), management (priority 5), risk factors (priority 9) and diagnostics (priority 10). The broad range underscores the complexity of challenges faced by individuals living with back pain, their healthcare providers and researchers.

To the best of our knowledge, no previous PSP has focused on the broad population of individuals with back pain. Only a few PSPs focused on specific back-related diagnoses, such as spinal cord injury and scoliosis, have been published [19–22]. Thus, direct comparability of these results with other studies is limited. Nevertheless, our findings are generally in accordance with those of a PSP conducted on chronic musculoskeletal disorders in Denmark [25]. Lyng et al. [25] also identified research priorities as improving knowledge implementation within the healthcare system, improving healthcare system organisation and consistency, increasing knowledge among patients, improving diagnostics and management, and expanding knowledge of risk factors. Furthermore, several of our findings are supported by research recommendations from the World Health Organization guidelines for the management of chronic low back pain in primary and community care settings [26]. These guidelines highlight key evidence gaps, including the need for implementation research, evaluation of interventions across population groups and healthcare settings, and strengthening of patient education. Also, a recent research synthesis by Pinto et al. [27] emphasises improving diagnostic accuracy, developing diagnostic risk models, and advancing classification systems to identify clinically meaningful subgroups with implications for management.

### Limitations and strengths

The main limitation of the present study is that the findings may have limited generalisability, as this PSP was conducted in a high-income setting targeting Norwegian-speaking participants living in Norway. Additionally, as with all PSPs, we relied on active and voluntary participation, which may have introduced self-selection bias [28,29]. The interim prioritisation survey had a disproportionate representation of individuals with lived experience of back pain, particularly females with long-lasting back pain (>10 years), while males and healthcare professionals were under-represented. It is unknown whether the sample obtained for the interim prioritisation survey accurately represents the broader population of individuals with lived experience of back pain and their healthcare providers, or whether certain preferences and priorities may be overrepresented [28,29]. Furthermore, participants in the focus groups and the final priority-setting workshop were predominantly native Norwegian speakers, which may have limited the inclusion of perspectives from individuals with different linguistic or cultural backgrounds. Nonetheless, the results of the interim prioritisation survey suggest significant similarities in research priorities between individuals with lived experience of back pain and healthcare professionals. Also, a more diverse sample of participants contributed when gathering evidence of uncertainty through focus group interviews, as well as when determining the top 10 research priorities during the final priority setting workshop. A second potential limitation is the use of focus group interviews to gather evidence of uncertainty. The JLA process aims to engage a broad range of respondents, and online surveys are typically being used to gather evidence of uncertainties, as they are expected to have a wide reach [17,30]. However, online surveys might lead to an overrepresentation of younger respondents and potentially exclude certain patient and healthcare practitioner groups with limited computer literacy [30,31], a situation that was deemed inappropriate for this PSP. It has previously been recommended to adapt the JLA ‘standard’ approach where necessary to maximise inclusivity [32]. Thus, informed by input from the Norwegian Spine and Back Pain Association regarding the method considered most appropriate to capture the perspectives of its members, we chose focus group interviews and purposive recruitment to ensure diversity in the characteristics of individuals with lived experience of back pain and healthcare practitioners [30]. A third potential limitation is that three of the healthcare professionals who participated in the focus group interviews, as well as one in the priority setting workshop, also held roles as researchers. Since the JLA process aims to gather uncertainties from end-users, researchers are typically excluded from the PSP. However, we chose to include these participants, clearly emphasizing that their contributions were intended to reflect the perspective of healthcare professionals. Their participation was prioritised, as it helped to shape research questions that were well-framed and likely to be understood as intended. A fourth potential limitation is that some of the core research questions, as well as some of the top 10 research priorities, are very broad. For example, “Which type of treatment measures for back pain work best for whom?” Some might argue that a research question like this is challenging for researchers to interpret or may encompass too many elements with no clear prioritisation between them [17,33]. However, many specific questions would also be difficult to prioritise and could risk diluting a key theme across multiple questions during the prioritisation process [17]. Therefore, the core research questions were intentionally broad in scope, and we encourage researchers to build upon them when developing projects. A fifth potential limitation relates to the scope of perspectives included. Conducted according to the JLA methodology, this study drew on input from individuals with lived experience and healthcare professionals, meaning that other relevant perspectives may not have been captured. Back pain is a complex condition influenced by multiple factors, including social and societal determinants [2], and system- and policy-level research has previously been highlighted as important [34]. Consequently, due to the study scope, research priorities related to broader structural or policy factors may be underrepresented. In summary, as with all PSPs, the process may be subject to bias at multiple stages, including participant recruitment, framing and interpretation of uncertainties, and consensus-based decision-making, which may have influenced the resulting priorities.

The main strength of the present study lies in its adherence to the JLA methodology [17], involving stakeholders at all stages of the process. Also, to the best of our knowledge, it is the first PSP to address research priorities related to the broad population of individuals with back pain. Identifying and prioritizing research questions among end-users is vital for facilitating and ensuring that research is both relevant and meaningful to those it aims to serve. We hope that research priorities identified in this study will guide future work in the field of back pain and influence researchers, policymakers, and funding bodies. In addition to disseminating the identified research priorities through academic publication, we will share the results via social media, at relevant conferences, and through patient organisations.

## Conclusion

In conclusion, this PSP has identified the top 10 research priorities for back pain, reflecting the perspectives of both individuals with lived experience of back pain and healthcare professionals. These results should not be considered a definitive list of research questions, but a guide to research areas that are meaningful to end-users. They provide a solid basis for future research focused on the needs and priorities of end-users.
