# Supplementary material for: Research priorities for back pain: Results from an adapted James Lind Alliance priority setting partnership
Source: PLoS One. 2026 Jul 13;21(7):e0353164. doi: 10.1371/journal.pone.0353164 (PMC13362125; doi:10.1371/journal.pone.0353164)
Supplement: Attachment — Submitted filename: Response to reviewers_JLA back pain.pdf [file pone.0353164.s004.pdf]

Author’s response to Editor and Reviewers

Dear Editor and Reviewers,

Thank you for the time and effort you have invested into reviewing our article. We greatly appreciate your valuable comments and recommendations. We have addressed each of your comments in the point-by-point response below and a revised version of the manuscript has been submitted. Changes are highlighted in yellow in the “*Revised Manuscript with Track Changes*”. We believe these revisions have enhanced the quality of the manuscript, and we hope they meet your expectations, making it suitable for publication in PLOS One.

Sincerely,

Rikke Munk Killingmo, on behalf of all authors

Editor

Your manuscript has been reviewed by two experts in the field. Both reviewers found the study to be important and the manuscript generally well written. However, they raised several substantive concerns that require attention before the manuscript can be considered for publication. I am therefore recommending Major Revision. In particular, I would like to draw your attention to the following key points, which both reviewers independently highlighted:

Comment 1: Methodological transparency: The manuscript should more clearly describe where the approach aligns with and diverges from the standard JLA methodology. The use of focus groups rather than surveys for generating research questions requires stronger justification.

Response: Thank you for this comment. We have aimed to be transparent regarding our method in the manuscript, but we agree that this could be presented more clearly. In response to your comment, we have added a dedicated section in the Materials and methods describing how the study deviated from the JLA Guidebook, including justification for our approach. We appreciate the suggestion and agree that this clarification is appropriate and valuable to include. Please refer to the Material and methods section or see below:

Deviations from the JLA Guidebook

This study deviated from the JLA Guidebook [17] in four ways. First, although a protocol outlining the aim, scope, and method was developed, it was not registered or published on the JLA website, as the study was conducted independently rather than as a formally registered JLA PSP. Second, no formal JLA adviser was involved due to independence and lack of funding for this service. Third, carers were not included as stakeholder group, as back pain is typically self-managed and rarely involves ongoing informal caregiving. Consequently, the perspectives of individuals with lived experience of back pain and healthcare professionals were prioritised. Fourth, uncertainties were identified through focus groups. While JLA PSPs typically use online surveys, focus groups and purposive recruitment were used to ensure participant diversity and enable in-depth discussion and collective reflection, revealing uncertainties that might not emerge through surveys.

Comment 2: Participant recruitment: Both reviewers noted the need for more detailed information on how patient representatives and clinicians were recruited, and how diversity was sought or achieved.

Response: We thank you for this comment and apologies for the lack of clarity regarding the recruitment process in the original manuscript. The Method section has now been revised to provide a more detailed description of how participants for the focus groups were recruited and the strategies employed to ensure diversity. Please refer to the Material and methods section or see below:

"Participants were recruited by the Norwegian Spine and Back Pain Association through its networks, in consultation with the project leaders (RMK and ATL) at Oslo University Hospital. Recruitment aimed to ensure diversity. For individuals with lived experience, variation in age, sex, place of residence, native language, and diagnosis (non-specific and specific, e.g., scoliosis) was sought. For healthcare professionals, recruitment aimed to include different professions, as well as representatives from primary and specialist healthcare settings, with variation in age and sex."

Regarding characteristics of the included participants in the focus groups and the achievement of diversity. For patient representatives, we recorded information on age, sex, and native language. For healthcare professionals, we recorded information on sex, native language, and profession. No additional characteristics were collected due to restrictions from the Data Protection Officer at Oslo University Hospital, which did not permit the collection of identifiable data. To improve transparency, we have now added information on native language in the Results section and included a corresponding limitation in the Discussion section, noting the potential underrepresentation of diverse perspectives. Please refer to the Result and Discussion section or se below:

.. 12 individuals with lived experience of back pain (median age 53 (range 22-77) years; 8 (67%) women; 10 (83%) native Norwegian speakers).."

"Furthermore, participants in the focus groups and the final priority-setting workshop were predominantly native Norwegian speakers, which may have limited the inclusion of perspectives from individuals with different linguistic or cultural backgrounds."

Comment 3: Evidence appraisal criteria: The process used to determine whether core questions had been "sufficiently addressed" by existing evidence needs to be more explicitly described. An appendix documenting the search strategy and appraisal rationale would strengthen transparency.

Response: Thank you for this important comment. We agree that transparency in how evidence is evaluated is crucial. As described in the Method section, all core research questions underwent an evidence review to determine whether they had been sufficiently addressed in the existing literature. PubMed was used as the primary search database, with priority given to high-quality evidence, preferably systematic reviews. The search strategy was guided by the wording of each individual question, with search terms derived from the key concepts of the question and supplemented by closely related or synonymous terms to ensure comprehensive coverage. If it was unclear whether a core research question had been adequately addressed, the question and relevant literature were reviewed by the Steering Committee to reach consensus. Questions were retained unless there was strong evidence indicating they had already been thoroughly addressed.

We did not employ a standardised search strategy suitable for inclusion as an appendix. Our approach aligns with the JLA methodology, which recommends evidence checking to be pragmatic and proportionate: the PSP should ensure that the questions put forward for prioritisation are broadly unanswered, and that decisions are made transparently (ref: James Lind Alliance Guidebook). We acknowledge that providing a standardised search strategy would have further enhanced transparency. However, we hope the reviewer also finds our approach consistent with the JLA guidance and acceptable in this context.

To further clarify our approach, we have now added the following description of the search strategy to the Methods section. Please refer to the Materials and methods section or se below:

"The search strategy was guided by the wording of each individual question, with search terms derived from the key concepts of the question and supplemented by closely related or synonymous terms to ensure comprehensive coverage."

Comment 4: Strengths and limitations: The discussion should more thoroughly address potential biases in recruitment, question formulation, and the final prioritisation workshop, including group dynamics among the nine participants.

Response: We thank the editor for this comment. We agree that PSP processes may be influenced by biases across all stages, including recruitment, question formulation, and consensus-based decision-making. While several potential sources of bias were already discussed, we have now added an explicit statement to acknowledge these influences more broadly and enhance clarity. Thank you. Please refer to the Discussion section or see below:

"In summary, as with all PSPs, the process may be subject to bias at multiple stages, including participant recruitment, framing and interpretation of uncertainties, and consensus-based decision-making, which may have influenced the resulting priorities."

Regarding group dynamics. We agree that reflecting on group dynamics and the management of differing views is important. Key aspects of facilitation and decision-making were described in the Method section (*"All discussions were chaired by the two project leaders (RMK and ATL) to ensure that no single group or individual dominated the decision-making process. The goal was to reach an agreement by consensus, with decisions made by majority vote if consensus could not be achieved").* To further clarify, we have added a brief statement in the Results section noting how discussions were conducted and experienced in practice. Specifically, we have included a statement noting that the discussions during the priority-setting workshop were conducted in a respectful and collaborative manner, with participants receptive to differing perspectives, and no indication of dominance by individuals or groups. Please refer to the result section or se below:

"… with no majority votes required. The discussions were conducted in a respectful and collaborative atmosphere, with participants actively engaging with and remaining receptive to differing perspectives. The two project leaders did not observe any dominance by individual participants or groups during the process."

Reviewer #1
Thank you for asking me to peer review this interesting, well written and well structured manuscript. I recommend minor revisions prior to publication and outline these below.

Comment 1: Introduction. I appreciate the concise and appropriate content here. However, I think it would be worth expanding a little on the heterogeneity associated with back pain, and adding a couple of lines acknowledging the varied trajectories of back pain - acute, subacute and chronic back pain, and also any data on inequalities in impact of back pain, if you have these for Norway or Europe perhaps?

Response: Thank you for your comment. We agree and have revised the beginning of the introduction to include the suggested additions. Please refer to the introduction or see below:

"Back pain is common across the lifespan [1, 2], and characterised by substantial heterogeneity in symptom presentation and clinical course. Symptoms range from mild to severe and disabling pain, and duration may vary from short episodes to long-lasting conditions [3]. The median lifetime prevalence of low back pain has been estimated at 42% globally [4]. While mild back pain tends to decline after midlife, severe and disabling back pain becomes more common with increasing age [5-7]."

Comment 2: Methods. The introductory paragraph states that the method is 'inspired by JLA' but reporting is to patient and public involvement guidance. I'm wondering why the study is not reported according to the priority setting partnership guidance on the Equator site?

Response: Thank you for this valuable comment. We agree that the study should be reported in accordance with the REporting guideline for PRIority SEtting of health research (REPRISE). The manuscript has now been revised to reflect this, and we have stated that the study follows the REPRISE guidelines. Please refer to the Material and methods section or see below:

"The PSP has been reported following the REporting guideline for PRIority SEtting of health research (REPRISE) [8]."

In addition, four adjustments have been made to the manuscript to align with the reporting recommendations:

#1) To more clearly state leadership and management structure, we have specified that the PSP was initiated by the Norwegian Spine and Back Pain Association and conducted in in collaboration with Oslo University hospital by adding *"…at Oslo University Hospital".* Refer to the Materials and methods section. (REPRISE no. 8, Describe the selection and structure of the leadership and management team)

#2) To clarify relevant experience within the project team, we have added: *"…patient representative with prior experience conducting a PSP using the JLA methodology (ATL)"*. Refer to Materials and methods section, Establishing a Steering Committee. (REPRISE no. 10, Describe any training or experience relevant to conducting priority setting).

#3) To provide clearer participant characteristics, we have added the following description of individuals with lived experience of back pain included in the focus groups: *"with lived experience of back pain (median age 53 (range 22-77) years; 8 (67%) women; 10 (83%) native Norwegian speakers)"*. Refer to Results section, *Gathering evidence uncertainties and summarising evidence uncertainties*. (REPRISE no. 15, Describe the characteristics of stakeholders).

#4) To address implementation and dissemination of priorities, we have added the following statement: *"We hope that research priorities identified in this study will guide future work in the field of back pain and influence researchers, policymakers, and funding bodies. In addition to disseminating the identified research priorities through academic publication, we will share the results via social media, at relevant conferences, and through patient organisations"*. Refer to Discussion section. (REPRISE no. 27, Describe how priorities were fed back to stakeholders and/or to the public; REPRISE no. 28, Outline the strategy or action plans for implementing priorities).

Comment 3: Methods. Further, I note the study diverges a little from the JLA guidance – it would be helpful to clarify where it is aligned to JLA, and where it is not. For example, I don't think a protocol was set or registered; I don't think carers were involved… It would be helpful to have more explanation to me as a reviewer, and also within the text. I think potentially the most notable divergence is the generation of knowledge gaps/research topics by a relatively small set of focus groups, and not surveys – and you may well have good reasons, but they need more explanation and justification. This point is discussed under limitations on p24 (of the pdf manuscript that I have) but I think we need more earlier. I find it hard to see the as justification for a small number involved in setting questions *instead of* a survey. I'm looking at the JLA guidebook 2021 https://nihr.widen.net/s/zrmtvrknlk/jla-guidebook-version-10-march-2021 and on page 34 it does say focus groups can occur 'as well as via a survey.' I think we may understand the method as an adapted JLA approach or PSP more broadly. I think this point is important, as once the paper is published, I may wish to share it with colleagues, but I wouldn't want them to think that it is necessarily JLA methodology/ for your paper to set a precedent. Broad generation of questions is surely important in JLA. Please do either correct my knowledge here, or address this issue in the manuscript.

Response: As noted, this study diverges slightly from the JLA guidance. We have aimed to be transparent regarding our method in the manuscript, but we agree that this could be presented more clearly. In response to your comment, we have added a dedicated section in the Materials and methods describing how the study deviated from the JLA Guidebook, including justification for our approach. We appreciate the suggestion and agree that this clarification is appropriate and valuable to include. Please refer to the Material and methods section or see below:

Deviations from the JLA Guidebook
This study deviated from the JLA Guidebook [17] in four ways. First, although a protocol outlining the aim, scope, and method was developed, it was not registered or published on the JLA website, as the study was conducted independently rather than as a formally registered JLA PSP. Second, no formal JLA adviser was

involved due to independence and lack of funding for this service. Third, carers were not included as stakeholder group, as back pain is typically self-managed and rarely involves ongoing informal caregiving. Consequently, the perspectives of individuals with lived experience of back pain and healthcare professionals were prioritised. Fourth, uncertainties were identified through focus groups. While JLA PSPs typically use online surveys, focus groups and purposive recruitment were used to ensure participant diversity and enable in-depth discussion and collective reflection, revealing uncertainties that might not emerge through surveys.

Comment 4: Methods. Recruitment for the focus groups – how did this occur exactly? This is obviously influential to the findings, so I think we need more detail on this for transparency.

Response: We agree and apologies for not clearly reporting this information in the original manuscript. We have now revised the text to provide a more detailed description of the recruitment process. Please refer to the Material and methods section or see below:

"Participants were recruited by the Norwegian Spine and Back Pain Association through its networks, in consultation with the project leaders (RMK and ATL) at Oslo University Hospital. Recruitment aimed to ensure diversity. For individuals with lived experience, variation in age, sex, place of residence, native language, and diagnosis (non-specific and specific, e.g., scoliosis) was sought. For healthcare professionals, recruitment aimed to include different professions, as well as representatives from primary and specialist healthcare settings, with variation in age and sex."

Comment 5: Methods. Setting the scope – I know that JLA is for health and healthcare research but there is rationale in the broad literature to also view back pain disability as influenced by social/political factors/structures such as workplace adaptations/interventions (or lack of), social security that addresses needs, broader policy responses, sources of marginalisation. It might be worth mentioning this within scope and then addressing the issue in the discussion. 'Back pain' is quite different to a more specific 'disease'.

Response: Thank you for this insightful comment. As noted, this study drew on input from individuals with lived experience and healthcare professionals, meaning that other relevant perspectives may not have been captured. We agree that back pain is influenced by broader social and policy factors beyond healthcare, and we appreciate the suggestion to acknowledge in the discussion that research priorities related to broader structural or policy factors may be underrepresented due to the study scope. Please refer to the Discussion section or se below:

"A fifth potential limitation relates to the scope of perspectives included. Conducted according to the JLA methodology, this study drew on input from individuals with lived experience and healthcare professionals, meaning that other relevant perspectives may not have been captured. Back pain is a complex condition influenced by multiple factors, including social and societal determinants [2], and system- and policy-level research has previously been highlighted as important [32]. Consequently, due to the study scope, research priorities related to broader structural or policy factors may be underrepresented."

Comment 6: Results. These are interesting and a little exciting! It's interesting to see so many with knowledge mobilisation/implementation roots. Were there any issues with language translation e.g. the term 'tool' occurred quite often? The English language can require a lot of context (I am sorry I do not know Norwegian) - were there any assumptions underpinning the meaning of 'tool' about what that was? Also is the term 'non-specific' being contested, or is something lost in translation?

Response: We agree that it is interesting to see so many priorities with knowledge mobilisation and implementation roots. The term *"tool"* was used six times across the top 25 ranked core research questions from the interim prioritisation survey, always as a translation of the Norwegian word *"hjelpeverktøy"*. In all cases, "*tool"* refers broadly to any resource, instrument, or aid intended to support the assessment, diagnosis, management, or self-management of back pain. During the focus group interviews, there was some discussion about whether to provide a more specific definition of *"tool"*. However, it was decided to keep the

term broad and avoid giving specific guidance. Accordingly, no additional assumptions were made regarding its meaning. The term *"non-specific" back pain* was also discussed during the focus group interviews. It was used consistently to refer to back pain not attributable to neurological involvement or a specific pathological cause. There is, of course, always a risk that some nuances may be lost in translation. However, we believe the translation was conducted carefully and appropriately. It was carried out by the lead author in close collaboration with the full author group, which included experienced researchers familiar with terminology in the field of back pain, as well as native speakers of both Norwegian and English.

No changes have been made.

Comment 7: Discussion. Within the summary line I think it would be worth inserting 'in Norway' just for clarity – you could also do this elsewhere.

Response: Thank you for your comment. We have included "in Norway" to improve clarity. Please refer to the Discussion section or see below:

"…and healthcare professionals in Norway."

Comment 8: Discussion. You might find more to discuss if also comparing with other literature and research recommendations made in guidelines for back pain.

Response: Thank you for this helpful suggestion. In response, we have expanded the discussion to contextualise our findings within existing literature and guideline-based research recommendations. Specifically, we have incorporated comparisons with the World Health Organization guidelines for chronic low back pain and a recent research synthesis by Pinto et al. This strengthens the interpretation and overall relevance of our findings. Please refer to the Discussion section or see below:

"Furthermore, several of our findings are supported by research recommendations from the World Health Organization guidelines for the management of chronic low back pain in primary and community care settings [26]. These guidelines highlight key evidence gaps, including the need for implementation research, evaluation of interventions across population groups and healthcare settings, and strengthening of patient education. Also, a recent research synthesis by Pinto et al. [27] emphasises improving diagnostic accuracy, developing diagnostic risk models, and advancing classification systems to identify clinically meaningful subgroups with implications for management."

Comment 9: Discussion. Would be good to have a subheading for strengths and limitations.

Response: Of course, we have now added this subheading to the Discussion section. Please refer to the Discussion section.

Comment 10: Discussion. I would contest the line 'Despite best efforts to engage with all stakeholders' – I think that given the broad nature and high prevalence of back pain, more organisations including those not dedicated to only back pain, could have facilitated involvement. I would be happy if you removed that phrase, but you could also address that issue more in the text.

Response: Thank you for this comment. We agree and have now removed the sentence "Despite best efforts to engage with all stakeholders" from the text, as the discussion reads clearly without it. No additional text was added; we simply removed the suggested line. Please refer to the Discussion section or se below:

"..which may have introduced self-selection bias [26, 27]. The interim prioritisation survey.."

Comment 11: Discussion. I agree with the issue you raise on representation – also you did not collect data on ethnicity or socioeconomic status – perhaps a recommendation for future work?

Response: Thank you for this important comment. We agree that representation is a key consideration. We collected information on native language (native Norwegian speaker vs. non-native) for participants in the focus groups and the priority-setting workshop. In the focus groups, 10 out of 12 individuals with lived experience of back pain were native Norwegian speakers, and all healthcare professionals were native Norwegian speakers. All participants in the final priority-setting workshop were also native Norwegian speakers. We did not collect data on ethnicity or socioeconomic status in the interim prioritisation survey due to restrictions from the Data Protection Officer at Oslo University Hospital, which did not permit the collection of identifiable data. To improve transparency, we have now added information on native language in the Results section and included a corresponding limitation in the Discussion section, noting the potential underrepresentation of diverse perspectives. Please refer to the Result and Discussion section or se below:

.. 12 individuals with lived experience of back pain (median age 53 (range 22-77) years; 8 (67%) women; 10 (83%) native Norwegian speakers)..”

“Furthermore, participants in the focus groups and the final priority-setting workshop were predominantly native Norwegian speakers, which may have limited the inclusion of perspectives from individuals with different linguistic or cultural backgrounds.”

Comment 12: Discussion. I’m not sure I agree that the fourth limitation given is a limitation as such – it’s just the way things are. I would be happy for you to remove it, or discuss it as a point that is not a limitation – maybe see what the other reviewer, or editor, thinks?

Response: Thank you for this thoughtful comment. We agree that the fourth point may not constitute a limitation as such and may be more appropriately framed as a consideration. However, we have chosen to retain it as a *potential* limitation, as previous feedback (including from stakeholders and researchers) has suggested that the broad scope of some of the research questions may be perceived as challenging for researchers to interpret and operationalise. As neither the other reviewer nor the editor has commented on this issue, we hope it is acceptable to retain the text in its original form.

No changes have been made.

Comment 13: Discussion. This is an optional point, but I would consider re-prioritising some of the space in the abstract, and expanding the conclusion, towards the main categories of findings including the very interesting knowledge implementation/mobilisation questions.

Response: We agree that it would be valuable to expand the conclusion of the abstract to also reflect the main categories of findings. This was carefully considered during the writing process. However, due to the 300-word limit, including this detail would have required the removal of essential information regarding the background, aim, method, and results. We therefore appreciate that this point is optional and hope it is acceptable to retain the abstract in its current form.

No changes have been made.

Reviewer #2

Overall, this is an important and well-conducted study, and the manuscript presents a generally clear and methodologically sound process. Below I provide several comments and suggestions aimed at improving clarity, transparency, and interpretability of the work.

Comment 1. Abstract: Line 47: The statement that none of the core questions had been "sufficiently addressed" is unclear without an explanation of how "sufficiently" was defined. Providing a short description of the criteria (e.g., type of evidence required, reliance on existing systematic reviews, or methodological thresholds) would strengthen transparency.

Response: Thank you for this comment. We agree that clarifying how "sufficiently addressed" was determined strengthens transparency. To address this while remaining within the 300-word limit, we have added a brief description in the abstract, noting that core questions were evaluated with priority given to high-quality evidence, preferably systematic reviews. Please refer to the Abstract or se below:

"…addressed by existing evidence (prioritising high-quality evidence, preferably systematic reviews)."

Comment 2. Abstract: The abstract reports only the top-ranked research priority. Including a brief indication of the major thematic categories represented across the top 10 (e.g., lifestyle factors, healthcare organisation, diagnostics, prevention, patient-centred care) would make the abstract more informative without compromising conciseness.

Response: Thank you for this comment. We agree that including the main thematic categories represented across the top 10 research priorities would enhance the informativeness of the abstract. This was carefully considered during the writing process. However, due to the 300-word limit, incorporating this information would have required the removal of essential information regarding the background, aim, method, and results. We therefore hope it is acceptable to retain the abstract in its current form, and that interested readers will refer to the full manuscript for a more detailed presentation of the findings.

No action done.

Comment 3. Introduction: The introduction could more clearly articulate why end-user involvement is especially important in back-pain research. Given the condition's subjective symptom profile, heterogeneous trajectories, and psychosocial influences, priorities set without patient involvement risk overlooking concerns most relevant to those living with back pain.

Response: Thank you for this insightful comment. We agree that it is important to articulate why end-user involvement is critical in back pain research. In response, we have revised the introduction. Please refer to the Introduction section or se below:

"…preferences of end-users [15]. Back pain is a complex condition, influenced by multiple factors and characterised by substantial heterogeneity in symptoms and clinical course [2]. Therefore, research priorities established without end-user involvement risk overlooking issues most relevant to those living with the condition and their healthcare providers."

Comment 4. Methods: More information is needed on how patient representatives were recruited. Clarifying whether recruitment was open, purposive, clinically mediated, or targeted would help readers assess representativeness and potential selection biases.
Comment 5. Methods: Relatedly, the manuscript would benefit from a clearer description of how diversity among both patients and clinicians was sought or achieved (e.g., variation in age, sex, geography, clinical background, diagnostic history).

Response: We thank you for these comments and apologies for not clearly reporting the recruitment process in the original manuscript. We have now revised the Method section to provide a more detailed description of how participants for the focus groups were recruited. Please refer to the Material and methods section or see below:

"Participants were recruited by the Norwegian Spine and Back Pain Association through its networks, in consultation with the project leaders (RMK and ATL) at Oslo University Hospital. Recruitment aimed to ensure diversity. For individuals with lived experience, variation in age, sex, place of residence, native language, and diagnosis (non-specific and specific, e.g., scoliosis) was sought. For healthcare professionals, recruitment aimed to include different professions, as well as representatives from primary and specialist healthcare settings, with variation in age and sex."

Regarding characteristics of the included participants in the focus groups. For patient representatives, we recorded information on age, sex, and native language. For healthcare professionals, we recorded information on sex, native language, and profession. No additional characteristics were collected due to restrictions from the Data Protection Officer at Oslo University Hospital, which did not permit the collection of identifiable data. To improve transparency, we have now added information on native language in the Results section and included a corresponding limitation in the Discussion section, noting the potential underrepresentation of diverse perspectives. Please refer to the Result and Discussion section or se below:

.. 12 individuals with lived experience of back pain (median age 53 (range 22-77) years; 8 (67%) women; 10 (83%) native Norwegian speakers).."

"Furthermore, participants in the focus groups and the final priority-setting workshop were predominantly native Norwegian speakers, which may have limited the inclusion of perspectives from individuals with different linguistic or cultural backgrounds."

Comment 6. Methods: Although the manuscript explains that evidence was evaluated to determine whether each core question had been "sufficiently addressed," this process remains somewhat opaque. Including an appendix with the search strategy, screening approach, and rationale for retaining or excluding each core question would greatly enhance methodological transparency.

Response: Thank you for this important comment. We agree that transparency in how evidence is evaluated is crucial. As described in the Method section, all core research questions underwent an evidence review to determine whether they had been sufficiently addressed in the existing literature. PubMed was used as the primary search database, with priority given to high-quality evidence, preferably systematic reviews. The search strategy was guided by the wording of each individual question, with search terms derived from the key concepts of the question and supplemented by closely related or synonymous terms to ensure comprehensive coverage. If it was unclear whether a core research question had been adequately addressed, the question and relevant literature were reviewed by the Steering Committee to reach consensus. Questions were retained unless there was strong evidence indicating they had already been thoroughly addressed.

We did not employ a standardised search strategy suitable for inclusion as an appendix. Our approach aligns with the JLA methodology, which recommends evidence checking to be pragmatic and proportionate: the PSP should ensure that the questions put forward for prioritisation are broadly unanswered, and that decisions are made transparently (ref: James Lind Alliance Guidebook). We acknowledge that providing a standardised search strategy would have further enhanced transparency. However, we hope the reviewer also finds our approach consistent with the JLA guidance and acceptable in this context.

To further clarify our approach, we have now added the following description of the search strategy to the Methods section. Please refer to the Materials and methods section or se below:

"The search strategy was guided by the wording of each individual question, with search terms derived from the key concepts of the question and supplemented by closely related or synonymous terms to ensure comprehensive coverage."

Comment 7. Results: Some results raise questions about how evidence sufficiency was judged. For example, the classification of "What types of physical activity and exercise are appropriate for individuals with back pain?" as insufficiently answered may appear surprising given the extensive literature showing no clear superiority of specific exercise modalities. Again, an appendix documenting the evidence appraisal would resolve this and strengthen transparency.

Response: We agree and acknowledge that current literature shows no clear superiority of specific exercise modalities for individuals with back pain. Nonetheless, aware of this literature, the steering committee retained the question "What types of physical activity and exercise are appropriate for individuals with back pain?" as the evidence base may evolve with future research. Moreover, the committee argued that this question continues to be of interest to both individuals with lived experience of back pain and healthcare professionals, indicating the ongoing relevance of further research in this area.

No action needed.

Comment 8. Results: One of the top 10 priorities concerns scoliosis ("risk of requiring spinal surgery"), which appears diagnostically narrower than the general back-pain population described in the manuscript. It would be helpful to clarify why this topic remained within scope and how it aligns with the PSP's stated population definition.

Response: Yes, one of the top 10 priorities relates to scoliosis and is diagnostically narrower than the other priorities on the list. The scope of the PSP was intentionally defined broadly by the steering committee to include all types of back pain requiring treatment or associated with disability, without excluding specific diagnoses. During the focus group phase, several uncertainties related to scoliosis were proposed. Some progressed through the interim prioritisation survey and one was ultimately retained in the final top 10 during the priority-setting workshop. Its inclusion therefore reflects the priorities identified by participants within the defined scope of the PSP.

No action needed.

Comment 9. Discussion: Given that the final top-10 workshop involved nine participants, the Discussion could benefit from a short reflection on potential group dynamics, how differing views were managed, and what steps were taken to minimise dominance effects.

Response: Thank you for this comment. We agree that reflecting on group dynamics and the management of differing views is important. While key aspects of facilitation and decision-making were already described in the Method section ("*All discussions were chaired by the two project leaders (RMK and ATL) to ensure that no single group or individual dominated the decision-making process. The goal was to reach an agreement by consensus, with decisions made by majority vote if consensus could not be achieved*"), we have now added a brief clarification in the Results section to further describe how discussions were conducted and experienced in practice. Specifically, we have included a statement noting that the discussions during the priority-setting workshop were conducted in a respectful and collaborative manner, with participants receptive to differing perspectives, and no indication of dominance by individuals or groups. Please refer to the result section or se below:

"… with no majority votes required. The discussions were conducted in a respectful and collaborative atmosphere, with participants actively engaging with and remaining receptive to differing perspectives. The two project leaders did not observe any dominance by individual participants or groups during the process."

Comment 10. Discussion: The Discussion could include a more explicit acknowledgement that PSP processes can be influenced by various forms of bias (e.g., in participant recruitment, question formulation, and decision-making stages).

Response: We agree that PSP processes may be influenced by various forms of bias across all stages, including recruitment, question formulation, and consensus-based decision-making. While several potential sources of bias were already discussed, we have now added a brief, explicit statement to acknowledge these influences more broadly and enhance clarity. Thank you. Please refer to the Discussion section or see below:

"In summary, as with all PSPs, the process may be subject to bias at multiple stages, including participant recruitment, framing and interpretation of uncertainties, and consensus-based decision-making, which may have influenced the resulting priorities."
