# Supplementary material for: Research priorities for back pain: Results from an adapted James Lind Alliance priority setting partnership
Source: PLoS One. 2026 Jul 13;21(7):e0353164. doi: 10.1371/journal.pone.0353164 (PMC13362125; doi:10.1371/journal.pone.0353164)
Supplement: Attachment — Submitted filename: Response to reviewer resubmisison JLA back pain.pdf [file pone.0353164.s005.pdf]

Author’s response to Editor and Reviewers

Dear Editor and Reviewers,

Thank you for the time and effort you have invested into re-reviewing our article. We greatly appreciate your valuable comments and recommendations. We have addressed each of your comments in the point-by-point response below and a revised version of the manuscript has been submitted. Changes are highlighted in yellow in the “*Revised Manuscript with Track Changes*”. We believe these revisions have enhanced the quality of the manuscript, and we hope they meet your expectations, making it suitable for publication in PLOS One.

Sincerely,

Rikke Munk Killingmo, on behalf of all authors

Reviewer #1
Thank you for asking me to review the revised manuscript which has improved and there is a more transparent methods section. I still have some concerns that are mainly focused on what the methodological framing of the study is – I do not find it to be a JLA study, rather a looser PSP. With some reframing in the manuscript, I think the study could be published as a PSP, but not as a JLA. The authors now note some of the divergent points in the manuscript and I have comments on some of these:

Comment 1: There is a divergence between the PSP study in the manuscript and that of a JLA in generating potential research questions through surveys – these can be paper, online etc., and using focus groups additionally, and this is not fully reflected in the manuscript as a major methodological point.

Response: Thank you for this comment. We agree that the use of focus groups instead of surveys represents an important methodological deviation from the traditional JLA PSP process.

In response to the previous review round, we added a dedicated section in the Materials and methods section describing how the study deviates from the JLA Guidebook. In this section, we clarify that uncertainties were identified through focus groups rather than surveys, and that this approach was chosen to ensure participant diversity and facilitate in-depth discussion and collective reflection: *“…Fourth, uncertainties were identified through focus groups. While JLA PSPs typically use online surveys, focus groups and purposive recruitment were used to ensure participant diversity and enable in-depth discussion and collective reflection, revealing uncertainties that might not emerge through surveys.”* The decision to adopt this approach was also informed by input from the Norwegian Spine and Back Pain Association regarding the method considered most appropriate to capture the perspectives of its members. This methodological choice is addressed as a potential limitation in the Discussion section.

To further address the reviewer’s concern, we have now explicitly emphasised in the manuscript that the use of focus groups rather than surveys constitutes a methodological adaptation of the traditional JLA PSP approach. Please refer to the Material and methods section or see below:

“Thus, the use of focus groups rather than surveys constituted a deliberate adaptation of the traditional JLA PSP approach.”

In the Discussion section, we further clarify that this decision was informed by input from the Norwegian Spine and Back Pain Association. Please refer to the Discussion section or see below:

"Thus, informed by input from the Norwegian Spine and Back Pain Association regarding the method considered most appropriate to capture the perspectives of its members, we chose focus group interviews .."

Comment 2: I am surprised to see the justification for not including informal carers as because they are rarely involved – this is contradicted by claims of the disabling nature of back pain and evidence on severe/high impact pain (and by my own clinical and research experience – may be a blind spot of this research team). At the minimum, a reference should be given here for that claim.

Response: Thank you for this important comment. We acknowledge that the original wording may have understated the role of informal caregiving in back pain management across different levels of severity.

We have therefore revised the sentence to better align with the broader literature on back pain. In particular, we have avoided implying that informal caregiving is absent and instead clarified that it is not typically a central component of routine management in the majority of cases. We have also added a supporting reference (*Hartvigsen et al. 2018, What low back pain is and why we need to pay attention).* Please refer to the Material and methods section or see below.

"Third, carers were not included as stakeholder group, as back pain is typically managed in primary care and largely self-managed, with informal caregiving not being a central component of routine management in most cases [2]."

Comment 3: The points about the recommended research priorities made by reviewer 2 also show divergence from a JLA approach. I am surprised that the authors haven't taken this on more in their revisions. I am not going to go into all of the divergent points, but this is why I think it is better to understand the study as a looser 'PSP.' I can see this research team have a similar study published as a JLA approach https://link.springer.com/article/10.1186/s41927-025-00588-7 and it is likely to be a contested point. I wonder if it would be best for the editor to contact the JLA directly with this query: https://www.jla.nihr.ac.uk/

Response: Thank you for this thoughtful comment. We appreciate the reviewer's reflection on the degree of alignment between our study and the standard JLA PSP methodology.

We acknowledge that some aspects of our approach represent contextual adaptations of the JLA methodology, particularly regarding recruitment procedures and the use of focus groups for identifying uncertainties. However, these adaptations were undertaken in accordance with established JLA guidance, which allows methodological flexibility to ensure inclusivity and feasibility within specific contexts. Importantly, the core principles of the JLA PSP methodology were maintained throughout the study, including stakeholder involvement, transparent prioritisation of uncertainties, and a structured consensus-based process involving both patients and healthcare professionals.

Methodological adaptations within JLA PSPs are not uncommon. For example, previous JLA PSPs have reported adaptations while still explicitly positioning their studies within the JLA framework (e.g. Apea et al., 2025 https://doi.org/10.1136/bmjopen-2024-086922; Harris et al., 2022 https://doi.org/10.1136/bmjopen-2021-055780; Lyng et al., 2022 https://doi.org/10.1515/sjpain-2022-0019).

We have carefully re-reviewed the manuscript in light of this comment. We have clarified the methodological deviations and their rationale in the Methods section, as well as their implications in the Discussion section. We consider that the manuscript already addresses concerns regarding transparency of methodological alignment. However, to further improve clarity and address the reviewer's concern regarding framing, we have revised the title of the manuscript to more explicitly reflect the adapted nature of our approach. Please refer to the Title or see below:

"Research priorities for back pain: results from an adapted James Lind Alliance priority setting partnership"

We believe this revised wording more accurately reflects the study methodology and its contextual adaptations, while maintaining clear alignment with the JLA framework. We thank the reviewer for the opportunity to clarify and strengthen the transparency of our reporting.

Reviewer #2

The authors have addressed all of my previous comments satisfactorily. The revisions have improved the clarity, transparency, and interpretability of the manuscript, particularly with regard to methodological decisions and their underlying rationale. The authors now clearly justify their choices, acknowledge potential limitations and sources of bias inherent to the methodology, and appropriately discuss the implications of these for interpretation of the findings. Overall, the manuscript presents a technically sound and well-reported study, and I consider it suitable for publication.
