# Supplementary material for: Research priorities for back pain: Results from an adapted James Lind Alliance priority setting partnership
Source: PLoS One. 2026 Jul 13;21(7):e0353164. doi: 10.1371/journal.pone.0353164 (PMC13362125; doi:10.1371/journal.pone.0353164)
Supplement: Attachment — Submitted filename: peer review PONE 13-12-25.docx [file pone.0353164.s001.docx]

Thank you for asking me to peer review this interesting, well written and well structured manuscript. I recommend minor revisions prior to publication and outline these below.

Introduction

I appreciate the concise and appropriate content here. However, I think it would be worth expanding a little on the heterogeneity associated with back pain, and adding a couple of lines acknowledging the varied trajectories of back pain - acute, subacute and chronic back pain, and also any data on inequalities in impact of back pain, if you have these for Norway or Europe perhaps?

Methods

The introductory paragraph states that the method is ‘inspired by JLA’ but reporting is to patient and public involvement guidance. I’m wondering why the study is not reported according to the priority setting partnership guidance on the Equator site? Further, I note the study diverges a little from the JLA guidance – it would be helpful to clarify where it is aligned to JLA, and where it is not. For example, I don’t think a protocol was set or registered; I don’t think carers were involved… It would be helpful to have more explanation to me as a reviewer, and also within the text.

I think potentially the most notable divergence is the generation of knowledge gaps/research topics by a relatively small set of focus groups, and not surveys – and you may well have good reasons, but they need more explanation and justification. This point is discussed under limitations on p24 (of the pdf manuscript that I have) but I think we need more earlier. I find it hard to see the as justification for a small number involved in setting questions *instead of* a survey. I’m looking at the JLA guidebook 2021 <https://nihr.widen.net/s/zrmtvrknlk/jla-guidebook-version-10-march-2021> and on page 34 it does say focus groups can occur ‘as well as via a survey.’ I think we may understand the method as an adapted JLA approach or PSP more broadly. I think this point is important, as once the paper is published, I may wish to share it with colleagues, but I wouldn’t want them to think that it is necessarily JLA methodology/ for your paper to set a precedent. Broad generation of questions is surely important in JLA. Please do either correct my knowledge here, or address this issue in the manuscript.

Recruitment for the focus groups – how did this occur exactly? This is obviously influential to the findings, so I think we need more detail on this for transparency.

Setting the scope – I know that JLA is for health and healthcare research but there is rationale in the broad literature to also view back pain disability as influenced by social/political factors/structures such as workplace adaptations/interventions (or lack of), social security that addresses needs, broader policy responses, sources of marginalisation. It might be worth mentioning this within scope and then addressing the issue in the discussion. ‘Back pain’ is quite different to a more specific ‘disease’.

Results

These are interesting and a little exciting! It’s interesting to see so many with knowledge mobilisation/implementation roots.

Were there any issues with language translation e.g. the term ‘tool’ occurred quite often? The English language can require a lot of context (I am sorry I do not know Norwegian) - were there any assumptions underpinning the meaning of ‘tool’ about what that was? Also is the term ‘non-specific’ being contested, or is something lost in translation?

Discussion

Within the summary line I think it would be worth inserting ‘in Norway’ just for clarity – you could also do this elsewhere.

You might find more to discuss if also comparing with other literature and research recommendations made in guidelines for back pain.

Would be good to have a subheading for strengths and limitations.

I would contest the line ‘Despite best efforts to engage with all stakeholders’ – I think that given the broad nature and high prevalence of back pain, more organisations including those not dedicated to only back pain, could have facilitated involvement. I would be happy if you removed that phrase, but you could also address that issue more in the text.

I agree with the issue you raise on representation – also you did not collect data on ethnicity or socioeconomic status – perhaps a recommendation for future work?

I’m not sure I agree that the fourth limitation given is a limitation as such – it’s just the way things are. I would be happy for you to remove it, or discuss it as a point that is not a limitation – maybe see what the other reviewer, or editor, thinks?

This is an optional point, but I would consider re-prioritising some of the space in the abstract, and expanding the conclusion, towards the main categories of findings including the very interesting knowledge implementation/mobilisation questions.
