# Supplementary material for: Research priorities for back pain: Results from an adapted James Lind Alliance priority setting partnership
Source: PLoS One. 2026 Jul 13;21(7):e0353164. doi: 10.1371/journal.pone.0353164 (PMC13362125; doi:10.1371/journal.pone.0353164)
Supplement: Attachment — Submitted filename: peer review PONE 17-5-26.docx [file pone.0353164.s003.docx]

May 2026

Thank you for asking me to review the revised manuscript which has improved and there is a more transparent methods section. I still have some concerns that are mainly focused on what the methodological framing of the study is – I do not find it to be a JLA study, rather a looser PSP. With some reframing in the manuscript, I think the study could be published as a PSP, but not as a JLA. The authors now note some of the divergent points in the manuscript and I have comments on some of these:

- There is a divergence between the PSP study in the manuscript and that of a JLA in generating potential research questions through surveys – these can be paper, online etc., and using focus groups additionally, and this is not fully reflected in the manuscript as a major methodological point.
- I am surprised to see the justification for not including informal carers as because they are rarely involved – this is contradicted by claims of the disabling nature of back pain and evidence on severe/high impact pain (and by my own clinical and research experience – may be a blind spot of this research team). At the minimum, a reference should be given here for that claim.
- The points about the recommended research priorities made by reviewer 2 also show divergence from a JLA approach. I am surprised that the authors haven’t taken this on more in their revisions.

I am not going to go into all of the divergent points, but this is why I think it is better to understand the study as a looser ‘PSP.’ I can see this research team have a similar study published as a JLA approach <https://link.springer.com/article/10.1186/s41927-025-00588-7> and it is likely to be a contested point. I wonder if it would be best for the editor to contact the JLA directly with this query: <https://www.jla.nihr.ac.uk/>
