# Supplementary material for: Research priorities for back pain: Results from an adapted James Lind Alliance priority setting partnership
Source: PLoS One. 2026 Jul 13;21(7):e0353164. doi: 10.1371/journal.pone.0353164 (PMC13362125; doi:10.1371/journal.pone.0353164)
Supplement: Peer review history

# Peer review: Decision Letter 0

**DOI:** 10.1371/journal.pone.0353164.r001

**Authors:** Alessandro Rodolico

9 Mar 2026

PONE-D-25-60540

Research priorities for back pain: results from a James Lind Alliance approach

PLOS One

Dear Dr. Killingmo,

Thank you for submitting your manuscript to PLOS ONE. After careful consideration, we feel that it has merit but does not fully meet PLOS ONE’s publication criteria as it currently stands. Therefore, we invite you to submit a revised version of the manuscript that addresses the points raised during the review process.

Your manuscript has been reviewed by two experts in the field. Both reviewers found the study to be important and the manuscript generally well written. However, they raised several substantive concerns that require attention before the manuscript can be considered for publication.

I am therefore recommending Major Revision.

In particular, I would like to draw your attention to the following key points, which both reviewers independently highlighted:

- Methodological transparency: The manuscript should more clearly describe where the approach aligns with and diverges from the standard JLA methodology. The use of focus groups rather than surveys for generating research questions requires stronger justification.

- Participant recruitment: Both reviewers noted the need for more detailed information on how patient representatives and clinicians were recruited, and how diversity was sought or achieved.

- Evidence appraisal criteria: The process used to determine whether core questions had been "sufficiently addressed" by existing evidence needs to be more explicitly described. An appendix documenting the search strategy and appraisal rationale would strengthen transparency.

- Strengths and limitations: The discussion should more thoroughly address potential biases in recruitment, question formulation, and the final prioritisation workshop, including group dynamics among the nine participants.

Please also address all additional comments raised by each reviewer individually.

When submitting your revision, please provide a point-by-point response to each reviewer's comments.

I look forward to receiving your revised manuscript.

Kind regards,

Alessandro Rodolico

Academic Editor, PLOS ONE

Please submit your revised manuscript by Apr 23 2026 11:59PM. If you will need more time than this to complete your revisions, please reply to this message or contact the journal office at plosone@plos.org. When you're ready to submit your revision, log on to https://www.editorialmanager.com/pone/ and select the 'Submissions Needing Revision' folder to locate your manuscript file.

Please include the following items when submitting your revised manuscript:

- A letter that responds to each point raised by the academic editor and reviewer(s). You should upload this letter as a separate file labeled 'Response to Reviewers'.
- A marked-up copy of your manuscript that highlights changes made to the original version. You should upload this as a separate file labeled 'Revised Manuscript with Track Changes'.
- An unmarked version of your revised paper without tracked changes. You should upload this as a separate file labeled 'Manuscript'.

If you would like to make changes to your financial disclosure, please include your updated statement in your cover letter. Guidelines for resubmitting your figure files are available below the reviewer comments at the end of this letter.

If applicable, we recommend that you deposit your laboratory protocols in protocols.io to enhance the reproducibility of your results. Protocols.io assigns your protocol its own identifier (DOI) so that it can be cited independently in the future. For instructions see: https://journals.plos.org/plosone/s/submission-guidelines#loc-laboratory-protocols. Additionally, PLOS ONE offers an option for publishing peer-reviewed Lab Protocol articles, which describe protocols hosted on protocols.io. Read more information on sharing protocols at https://plos.org/protocols?utm_medium=editorial-email&utm_source=authorletters&utm_campaign=protocols.

We look forward to receiving your revised manuscript.

Journal Requirements:

When submitting your revision, we need you to address these additional requirements.

1. Please ensure that your manuscript meets PLOS ONE's style requirements, including those for file naming. The PLOS ONE style templates can be found at

https://journals.plos.org/plosone/s/file?id=wjVg/PLOSOne_formatting_sample_main_body.pdf and

https://journals.plos.org/plosone/s/file?id=ba62/PLOSOne_formatting_sample_title_authors_affiliations.pdf

2.  We note that the grant information you provided in the ‘Funding Information’ and ‘Financial Disclosure’ sections do not match.

When you resubmit, please ensure that you provide the correct grant numbers for the awards you received for your study in the ‘Funding Information’ section.

3. Thank you for stating the following financial disclosure: [This study was supported by Foundation Dam (grant no. SDAM_UTV452311). Open access funding was provided by the Oslo Metropolitan University. Funding organisations had no part in the planning, performing, or reporting of the study.].

Please state what role the funders took in the study.  If the funders had no role, please state: ""The funders had no role in study design, data collection and analysis, decision to publish, or preparation of the manuscript.""

If this statement is not correct you must amend it as needed.

Please include this amended Role of Funder statement in your cover letter; we will change the online submission form on your behalf.

4. In the online submission form you indicate that your data is not available for proprietary reasons and have provided a contact point for accessing this data. Please note that your current contact point is a co-author on this manuscript. According to our Data Policy, the contact point must not be an author on the manuscript and must be an institutional contact, ideally not an individual. Please revise your data statement to a non-author institutional point of contact, such as a data access or ethics committee, and send this to us via return email. Please also include contact information for the third party organization, and please include the full citation of where the data can be found.

5. If the reviewer comments include a recommendation to cite specific previously published works, please review and evaluate these publications to determine whether they are relevant and should be cited. There is no requirement to cite these works unless the editor has indicated otherwise.

[Note: HTML markup is below. Please do not edit.]

Reviewers' comments:

Reviewer's Responses to Questions

**Comments to the Author**

1. Is the manuscript technically sound, and do the data support the conclusions?

The manuscript must describe a technically sound piece of scientific research with data that supports the conclusions. Experiments must have been conducted rigorously, with appropriate controls, replication, and sample sizes. The conclusions must be drawn appropriately based on the data presented.

Reviewer #1: Partly

Reviewer #2: Yes

**********

2. Has the statistical analysis been performed appropriately and rigorously?

Reviewer #1: N/A

Reviewer #2: Yes

**********

3. Have the authors made all data underlying the findings in their manuscript fully available?

The PLOS Data policy requires authors to make all data underlying the findings described in their manuscript fully available without restriction, with rare exception (please refer to the Data Availability Statement in the manuscript PDF file). The data should be provided as part of the manuscript or its supporting information, or deposited to a public repository. For example, in addition to summary statistics, the data points behind means, medians and variance measures should be available. If there are restrictions on publicly sharing data—e.g. participant privacy or use of data from a third party—those must be specified.

Reviewer #1: Yes

Reviewer #2: Yes

**********

4. Is the manuscript presented in an intelligible fashion and written in standard English?

PLOS ONE does not copyedit accepted manuscripts, so the language in submitted articles must be clear, correct, and unambiguous. Any typographical or grammatical errors should be corrected at revision, so please note any specific errors here.

Reviewer #1: Yes

Reviewer #2: Yes

**********

5. Review Comments to the Author

Please use the space provided to explain your answers to the questions above. You may also include additional comments for the author, including concerns about dual publication, research ethics, or publication ethics. (Please upload your review as an attachment if it exceeds 20,000 characters)

Reviewer #1: Comments are in the reviewer file that I uploaded - I cannot proceed without more text in this box so shall copy and text the file text.

Thank you for asking me to peer review this interesting, well written and well structured manuscript. I recommend minor revisions prior to publication and outline these below.

Introduction

I appreciate the concise and appropriate content here. However, I think it would be worth expanding a little on the heterogeneity associated with back pain, and adding a couple of lines acknowledging the varied trajectories of back pain - acute, subacute and chronic back pain, and also any data on inequalities in impact of back pain, if you have these for Norway or Europe perhaps?

Methods

The introductory paragraph states that the method is ‘inspired by JLA’ but reporting is to patient and public involvement guidance. I’m wondering why the study is not reported according to the priority setting partnership guidance on the Equator site? Further, I note the study diverges a little from the JLA guidance – it would be helpful to clarify where it is aligned to JLA, and where it is not. For example, I don’t think a protocol was set or registered; I don’t think carers were involved… It would be helpful to have more explanation to me as a reviewer, and also within the text.

I think potentially the most notable divergence is the generation of knowledge gaps/research topics by a relatively small set of focus groups, and not surveys – and you may well have good reasons, but they need more explanation and justification. This point is discussed under limitations on p24 (of the pdf manuscript that I have) but I think we need more earlier. I find it hard to see the as justification for a small number involved in setting questions instead of a survey. I’m looking at the JLA guidebook 2021 https://nihr.widen.net/s/zrmtvrknlk/jla-guidebook-version-10-march-2021 and on page 34 it does say focus groups can occur ‘as well as via a survey.’ I think we may understand the method as an adapted JLA approach or PSP more broadly. I think this point is important, as once the paper is published, I may wish to share it with colleagues, but I wouldn’t want them to think that it is necessarily JLA methodology/ for your paper to set a precedent. Broad generation of questions is surely important in JLA. Please do either correct my knowledge here, or address this issue in the manuscript.

Recruitment for the focus groups – how did this occur exactly? This is obviously influential to the findings, so I think we need more detail on this for transparency.

Setting the scope – I know that JLA is for health and healthcare research but there is rationale in the broad literature to also view back pain disability as influenced by social/political factors/structures such as workplace adaptations/interventions (or lack of), social security that addresses needs, broader policy responses, sources of marginalisation. It might be worth mentioning this within scope and then addressing the issue in the discussion. ‘Back pain’ is quite different to a more specific ‘disease’.

Results

These are interesting and a little exciting! It’s interesting to see so many with knowledge mobilisation/implementation roots.

Were there any issues with language translation e.g. the term ‘tool’ occurred quite often? The English language can require a lot of context (I am sorry I do not know Norwegian) - were there any assumptions underpinning the meaning of ‘tool’ about what that was? Also is the term ‘non-specific’ being contested, or is something lost in translation?

Discussion

Within the summary line I think it would be worth inserting ‘in Norway’ just for clarity – you could also do this elsewhere.

You might find more to discuss if also comparing with other literature and research recommendations made in guidelines for back pain.

Would be good to have a subheading for strengths and limitations.

I would contest the line ‘Despite best efforts to engage with all stakeholders’ – I think that given the broad nature and high prevalence of back pain, more organisations including those not dedicated to only back pain, could have facilitated involvement. I would be happy if you removed that phrase, but you could also address that issue more in the text.

I agree with the issue you raise on representation – also you did not collect data on ethnicity or socioeconomic status – perhaps a recommendation for future work?

I’m not sure I agree that the fourth limitation given is a limitation as such – it’s just the way things are. I would be happy for you to remove it, or discuss it as a point that is not a limitation – maybe see what the other reviewer, or editor, thinks?

This is an optional point, but I would consider re-prioritising some of the space in the abstract, and expanding the conclusion, towards the main categories of findings including the very interesting knowledge implementation/mobilisation questions.

Reviewer #2: Overall, this is an important and well‑conducted study, and the manuscript presents a generally clear and methodologically sound process. Below I provide several comments and suggestions aimed at improving clarity, transparency, and interpretability of the work.

Abstract:

Line 47: The statement that none of the core questions had been “sufficiently addressed” is unclear without an explanation of how “sufficiently” was defined. Providing a short description of the criteria (e.g., type of evidence required, reliance on existing systematic reviews, or methodological thresholds) would strengthen transparency.

Abstract:

The abstract reports only the top‑ranked research priority. Including a brief indication of the major thematic categories represented across the top 10 (e.g., lifestyle factors, healthcare organisation, diagnostics, prevention, patient‑centred care) would make the abstract more informative without compromising conciseness.

Introduction:

The introduction could more clearly articulate why end‑user involvement is especially important in back‑pain research. Given the condition’s subjective symptom profile, heterogeneous trajectories, and psychosocial influences, priorities set without patient involvement risk overlooking concerns most relevant to those living with back pain.

Methods:

More information is needed on how patient representatives were recruited. Clarifying whether recruitment was open, purposive, clinically mediated, or targeted would help readers assess representativeness and potential selection biases.

Methods:

Relatedly, the manuscript would benefit from a clearer description of how diversity among both patients and clinicians was sought or achieved (e.g., variation in age, sex, geography, clinical background, diagnostic history).

Methods:

Although the manuscript explains that evidence was evaluated to determine whether each core question had been “sufficiently addressed,” this process remains somewhat opaque. Including an appendix with the search strategy, screening approach, and rationale for retaining or excluding each core question would greatly enhance methodological transparency.

Results:

Some results raise questions about how evidence sufficiency was judged. For example, the classification of “What types of physical activity and exercise are appropriate for individuals with back pain?” as insufficiently answered may appear surprising given the extensive literature showing no clear superiority of specific exercise modalities. Again, an appendix documenting the evidence appraisal would resolve this and strengthen transparency.

Results:

One of the top 10 priorities concerns scoliosis (“risk of requiring spinal surgery”), which appears diagnostically narrower than the general back‑pain population described in the manuscript. It would be helpful to clarify why this topic remained within scope and how it aligns with the PSP’s stated population definition.

Discussion:

Given that the final top‑10 workshop involved nine participants, the Discussion could benefit from a short reflection on potential group dynamics, how differing views were managed, and what steps were taken to minimise dominance effects.

Discussion:

The Discussion could include a more explicit acknowledgement that PSP processes can be influenced by various forms of bias (e.g., in participant recruitment, question formulation, and decision‑making stages).

**********

6. PLOS authors have the option to publish the peer review history of their article (what does this mean?). If published, this will include your full peer review and any attached files.

If you choose “no”, your identity will remain anonymous but your review may still be made public.

**Do you want your identity to be public for this peer review?**  For information about this choice, including consent withdrawal, please see our Privacy Policy.

Reviewer #1: No

Reviewer #2: No

**********

[NOTE: If reviewer comments were submitted as an attachment file, they will be attached to this email and accessible via the submission site. Please log into your account, locate the manuscript record, and check for the action link "View Attachments". If this link does not appear, there are no attachment files.]

To ensure your figures meet our technical requirements, please review our figure guidelines: https://journals.plos.org/plosone/s/figures

You may also use PLOS’s free figure tool, NAAS, to help you prepare publication quality figures: https://journals.plos.org/plosone/s/figures#loc-tools-for-figure-preparation.

NAAS will assess whether your figures meet our technical requirements by comparing each figure against our figure specifications.

Attachment Submitted filename: peer review PONE 13-12-25.docx

# Author comment: Author response to Decision Letter 1

**DOI:** 10.1371/journal.pone.0353164.r002

30 Mar 2026

Dear Editor and Reviewers,

Thank you for the time and effort you have invested into reviewing our article. We greatly appreciate your valuable comments and recommendations. We have addressed each of your comments in the point-by-point response below and a revised version of the manuscript has been submitted. Changes are highlighted in yellow in the “Revised Manuscript with Track Changes”. We believe these revisions have enhanced the quality of the manuscript, and we hope they meet your expectations, making it suitable for publication in PLOS One.

Sincerely,

Rikke Munk Killingmo, on behalf of all authors

Editor

Your manuscript has been reviewed by two experts in the field. Both reviewers found the study to be important and the manuscript generally well written. However, they raised several substantive concerns that require attention before the manuscript can be considered for publication. I am therefore recommending Major Revision. In particular, I would like to draw your attention to the following key points, which both reviewers independently highlighted:

Comment 1: Methodological transparency: The manuscript should more clearly describe where the approach aligns with and diverges from the standard JLA methodology. The use of focus groups rather than surveys for generating research questions requires stronger justification.

Response: Thank you for this comment. We have aimed to be transparent regarding our method in the manuscript, but we agree that this could be presented more clearly. In response to your comment, we have added a dedicated section in the Materials and methods describing how the study deviated from the JLA Guidebook, including justification for our approach. We appreciate the suggestion and agree that this clarification is appropriate and valuable to include. Please refer to the Material and methods section or see below:

Deviations from the JLA Guidebook

This study deviated from the JLA Guidebook [17] in four ways. First, although a protocol outlining the aim, scope, and method was developed, it was not registered or published on the JLA website, as the study was conducted independently rather than as a formally registered JLA PSP. Second, no formal JLA adviser was involved due to independence and lack of funding for this service. Third, carers were not included as stakeholder group, as back pain is typically self-managed and rarely involves ongoing informal caregiving. Consequently, the perspectives of individuals with lived experience of back pain and healthcare professionals were prioritised. Fourth, uncertainties were identified through focus groups. While JLA PSPs typically use online surveys, focus groups and purposive recruitment were used to ensure participant diversity and enable in-depth discussion and collective reflection, revealing uncertainties that might not emerge through surveys.

Comment 2: Participant recruitment: Both reviewers noted the need for more detailed information on how patient representatives and clinicians were recruited, and how diversity was sought or achieved.

Response: We thank you for this comment and apologies for the lack of clarity regarding the recruitment process in the original manuscript. The Method section has now been revised to provide a more detailed description of how participants for the focus groups were recruited and the strategies employed to ensure diversity. Please refer to the Material and methods section or see below:

“Participants were recruited by the Norwegian Spine and Back Pain Association through its networks, in consultation with the project leaders (RMK and ATL) at Oslo University Hospital. Recruitment aimed to ensure diversity. For individuals with lived experience, variation in age, sex, place of residence, native language, and diagnosis (non-specific and specific, e.g., scoliosis) was sought. For healthcare professionals, recruitment aimed to include different professions, as well as representatives from primary and specialist healthcare settings, with variation in age and sex.”

Regarding characteristics of the included participants in the focus groups and the achievement of diversity. For patient representatives, we recorded information on age, sex, and native language. For healthcare professionals, we recorded information on sex, native language, and profession. No additional characteristics were collected due to restrictions from the Data Protection Officer at Oslo University Hospital, which did not permit the collection of identifiable data. To improve transparency, we have now added information on native language in the Results section and included a corresponding limitation in the Discussion section, noting the potential underrepresentation of diverse perspectives. Please refer to the Result and Discussion section or se below:

12 individuals with lived experience of back pain (median age 53 (range 22-77) years; 8 (67%) women; 10 (83%) native Norwegian speakers)..”

“Furthermore, participants in the focus groups and the final priority-setting workshop were predominantly native Norwegian speakers, which may have limited the inclusion of perspectives from individuals with different linguistic or cultural backgrounds.”

Comment 3: Evidence appraisal criteria: The process used to determine whether core questions had been "sufficiently addressed" by existing evidence needs to be more explicitly described. An appendix documenting the search strategy and appraisal rationale would strengthen transparency.

Response: Thank you for this important comment. We agree that transparency in how evidence is evaluated is crucial. As described in the Method section, all core research questions underwent an evidence review to determine whether they had been sufficiently addressed in the existing literature. PubMed was used as the primary search database, with priority given to high-quality evidence, preferably systematic reviews. The search strategy was guided by the wording of each individual question, with search terms derived from the key concepts of the question and supplemented by closely related or synonymous terms to ensure comprehensive coverage. If it was unclear whether a core research question had been adequately addressed, the question and relevant literature were reviewed by the Steering Committee to reach consensus. Questions were retained unless there was strong evidence indicating they had already been thoroughly addressed.

We did not employ a standardised search strategy suitable for inclusion as an appendix. Our approach aligns with the JLA methodology, which recommends evidence checking to be pragmatic and proportionate: the PSP should ensure that the questions put forward for prioritisation are broadly unanswered, and that decisions are made transparently (ref: James Lind Alliance Guidebook). We acknowledge that providing a standardised search strategy would have further enhanced transparency. However, we hope the reviewer also finds our approach consistent with the JLA guidance and acceptable in this context.

To further clarify our approach, we have now added the following description of the search strategy to the Methods section. Please refer to the Materials and methods section or se below:

“The search strategy was guided by the wording of each individual question, with search terms derived from the key concepts of the question and supplemented by closely related or synonymous terms to ensure comprehensive coverage.”

Comment 4: Strengths and limitations: The discussion should more thoroughly address potential biases in recruitment, question formulation, and the final prioritisation workshop, including group dynamics among the nine participants.

Response: We thank the editor for this comment. We agree that PSP processes may be influenced by biases across all stages, including recruitment, question formulation, and consensus-based decision-making. While several potential sources of bias were already discussed, we have now added an explicit statement to acknowledge these influences more broadly and enhance clarity. Thank you. Please refer to the Discussion section or see below:

“In summary, as with all PSPs, the process may be subject to bias at multiple stages, including participant recruitment, framing and interpretation of uncertainties, and consensus-based decision-making, which may have influenced the resulting priorities.”

Regarding group dynamics. We agree that reflecting on group dynamics and the management of differing views is important. Key aspects of facilitation and decision-making were described in the Method section (“All discussions were chaired by the two project leaders (RMK and ATL) to ensure that no single group or individual dominated the decision-making process. The goal was to reach an agreement by consensus, with decisions made by majority vote if consensus could not be achieved”). To further clarify, we have added a brief statement in the Results section noting how discussions were conducted and experienced in practice. Specifically, we have included a statement noting that the discussions during the priority-setting workshop were conducted in a respectful and collaborative manner, with participants receptive to differing perspectives, and no indication of dominance by individuals or groups. Please refer to the result section or se below:

“… with no majority votes required. The discussions were conducted in a respectful and collaborative atmosphere, with participants actively engaging with and remaining receptive to differing perspectives. The two project leaders did not observe any dominance by individual participants or groups during the process.”

Reviewer #1

Thank you for asking me to peer review this interesting, well written and well structured manuscript. I recommend minor revisions prior to publication and outline these below.

Comment 1: Introduction. I appreciate the concise and appropriate content here. However, I think it would be worth expanding a little on the heterogeneity associated with back pain, and adding a couple of lines acknowledging the varied trajectories of back pain - acute, subacute and chronic back pain, and also any data on inequalities in impact of back pain, if you have these for Norway or Europe perhaps?

Response: Thank you for your comment. We agree and have revised the beginning of the introduction to include the suggested additions. Please refer to the introduction or see below:

“Back pain is common across the lifespan [1, 2], and characterised by substantial heterogeneity in symptom presentation and clinical course. Symptoms range from mild to severe and disabling pain, and duration may vary from short episodes to long-lasting conditions [3]. The median lifetime prevalence of low back pain has been estimated at 42% globally [4]. While mild back pain tends to decline after midlife, severe and disabling back pain becomes more common with increasing age [5-7].”

Comment 2: Methods. The introductory paragraph states that the method is ‘inspired by JLA’ but reporting is to patient and public involvement guidance. I’m wondering why the study is not reported according to the priority setting partnership guidance on the Equator site?

Response: Thank you for this valuable comment. We agree that the study should be reported in accordance with the REporting guideline for PRIority SEtting of health research (REPRISE). The manuscript has now been revised to reflect this, and we have stated that the study follows the REPRISE guidelines. Please refer to the Material and methods section or see below:

“The PSP has been reported following the REporting guideline for PRIority SEtting of health research (REPRISE) [8].”

In addition, four adjustments have been made to the manuscript to align with the reporting recommendations:

#1) To more clearly state leadership and management structure, we have specified that the PSP was initiated by the Norwegian Spine and Back Pain Association and conducted in in collaboration with Oslo University hospital by adding “…at Oslo University Hospital”. Refer to the Materials and methods section. (REPRISE no. 8, Describe the selection and structure of the leadership and management team)

#2) To clarify relevant experience within the project team, we have added: “…patient representative with prior experience conducting a PSP using the JLA methodology (ATL)”. Refer to Materials and methods section, Establishing a Steering Committee. (REPRISE no. 10, Describe any training or experience relevant to conducting priority setting).

#3) To provide clearer participant characteristics, we have added the following description of individuals with lived experience of back pain included in the focus groups: “with lived experience of back pain (median age 53 (range 22-77) years; 8 (67%) women; 10 (83%) native Norwegian speakers)”. Refer to Results section, Gathering evidence uncertainties and summarising evidence uncertainties. (REPRISE no. 15, Describe the characteristics of stakeholders).

#4) To address implementation and dissemination of priorities, we have added the following statement: “We hope that research priorities identified in this study will guide future work in the field of back pain and influence researchers, policymakers, and funding bodies. In addition to disseminating the identified research priorities through academic publication, we will share the results via social media, at relevant conferences, and through patient organisations”. Refer to Discussion section. (REPRISE no. 27, Describe how priorities were fed back to stakeholders and/or to the public; REPRISE no. 28, Outline the strategy or action plans for implementing priorities).

Comment 3: Methods. Further, I note the study diverges a little from the JLA guidance – it would be helpful to clarify where it is aligned to JLA, and where it is not. For example, I don’t think a protocol was set or registered; I don’t think carers were involved… It would be helpful to have more explanation to me as a reviewer, and also within the text. I think potentially the most notable divergence is the generation of knowledge gaps/research topics by a relatively small set of focus groups, and not surveys – and you may well have good reasons, but they need more explanation and justification. This point is discussed under limitations on p24 (of the pdf manuscript that I have) but I think we need more earlier. I find it hard to see the as justification for a small number involved in setting questions instead of a survey. I’m looking at the JLA guidebook 2021 https://nihr.widen.net/s/zrmtvrknlk/jla-guidebook-version-10-march-2021 and on page 34 it does say focus groups can occur ‘as well as via a survey.’ I think we may understand the method as an adapted JLA approach or PSP more broadly. I think this point is important, as once the paper is published, I may wish to share it with colleagues, but I wouldn’t want them to think that it is necessarily JLA methodology/ for your paper to set a precedent. Broad generation of questions is surely important in JLA. Please do either correct my knowledge here, or address this issue in the manuscript.

Response: As noted, this study diverges slightly from the JLA guidance. We have aimed to be transparent regarding our method in the manuscript, but we agree that this could be presented more clearly. In response to your comment, we have added a dedicated section in the Materials and methods describing how the study deviated from the JLA Guidebook, including justification for our approach. We appreciate the suggestion and agree that this clarification is appropriate and valuable to include. Please refer to the Material and methods section or see below:

Deviations from the JLA Guidebook

This study deviated from the JLA Guidebook [17] in four ways. First, although a protocol outlining the aim, scope, and method was developed, it was not registered or published on the JLA website, as the study was conducted independently rather than as a formally registered JLA PSP. Second, no formal JLA adviser was involved due to independence and lack of funding for this service. Third, carers were not included as stakeholder group, as back pain is typically self-managed and rarely involves ongoing informal c

Attachment Submitted filename: Response to reviewers_JLA back pain.pdf

# Peer review: Decision Letter 1

**DOI:** 10.1371/journal.pone.0353164.r003

**Authors:** Alessandro Rodolico, Vincenzo De Luca

17 May 2026

PONE-D-25-60540R1

Research priorities for back pain: results from a James Lind Alliance approach

PLOS One

Dear Dr. Killingmo,

Thank you for submitting your manuscript to PLOS ONE. After careful consideration, we feel that it has merit but does not fully meet PLOS ONE’s publication criteria as it currently stands. Therefore, we invite you to submit a revised version of the manuscript that addresses the points raised during the review process.

Please submit your revised manuscript by Jul 01 2026 11:59PM. If you will need more time than this to complete your revisions, please reply to this message or contact the journal office at plosone@plos.org. When you're ready to submit your revision, log on to https://www.editorialmanager.com/pone/ and select the 'Submissions Needing Revision' folder to locate your manuscript file.

Please include the following items when submitting your revised manuscript:

- A letter that responds to each point raised by the academic editor and reviewer(s). You should upload this letter as a separate file labeled 'Response to Reviewers'.
- A marked-up copy of your manuscript that highlights changes made to the original version. You should upload this as a separate file labeled 'Revised Manuscript with Track Changes'.
- An unmarked version of your revised paper without tracked changes. You should upload this as a separate file labeled 'Manuscript'.

If you would like to make changes to your financial disclosure, please include your updated statement in your cover letter. Guidelines for resubmitting your figure files are available below the reviewer comments at the end of this letter.

If applicable, we recommend that you deposit your laboratory protocols in protocols.io to enhance the reproducibility of your results. Protocols.io assigns your protocol its own identifier (DOI) so that it can be cited independently in the future. For instructions see: https://journals.plos.org/plosone/s/submission-guidelines#loc-laboratory-protocols. Additionally, PLOS ONE offers an option for publishing peer-reviewed Lab Protocol articles, which describe protocols hosted on protocols.io. Read more information on sharing protocols at https://plos.org/protocols?utm_medium=editorial-email&utm_source=authorletters&utm_campaign=protocols.

As the corresponding author, your ORCID iD is verified in the submission system and will appear in the published article. PLOS supports the use of ORCID, and we encourage all coauthors to register for an ORCID iD and use it as well. Please encourage your coauthors to verify their ORCID iD within the submission system before final acceptance, as unverified ORCID iDs will not appear in the published article. *Only*  the individual author can complete the verification step; PLOS staff *cannot*  verify ORCID iDs on behalf of authors.

We look forward to receiving your revised manuscript.

Kind regards,

Vincenzo De Luca

Academic Editor

PLOS One

Journal Requirements:

If the reviewer comments include a recommendation to cite specific previously published works, please review and evaluate these publications to determine whether they are relevant and should be cited. There is no requirement to cite these works unless the editor has indicated otherwise.

Please review your reference list to ensure that it is complete and correct. If you have cited papers that have been retracted, please include the rationale for doing so in the manuscript text, or remove these references and replace them with relevant current references. Any changes to the reference list should be mentioned in the rebuttal letter that accompanies your revised manuscript. If you need to cite a retracted article, indicate the article’s retracted status in the References list and also include a citation and full reference for the retraction notice.

[Note: HTML markup is below. Please do not edit.]

Reviewers' comments:

Reviewer's Responses to Questions

**Comments to the Author**

1. If the authors have adequately addressed your comments raised in a previous round of review and you feel that this manuscript is now acceptable for publication, you may indicate that here to bypass the “Comments to the Author” section, enter your conflict of interest statement in the “Confidential to Editor” section, and submit your "Accept" recommendation.

Reviewer #1: (No Response)

Reviewer #2: All comments have been addressed

**********

2. Is the manuscript technically sound, and do the data support the conclusions?

The manuscript must describe a technically sound piece of scientific research with data that supports the conclusions. Experiments must have been conducted rigorously, with appropriate controls, replication, and sample sizes. The conclusions must be drawn appropriately based on the data presented.

Reviewer #1: Partly

Reviewer #2: Yes

**********

3. Has the statistical analysis been performed appropriately and rigorously?

Reviewer #1: N/A

Reviewer #2: N/A

**********

4. Have the authors made all data underlying the findings in their manuscript fully available?

The PLOS Data policy requires authors to make all data underlying the findings described in their manuscript fully available without restriction, with rare exception (please refer to the Data Availability Statement in the manuscript PDF file). The data should be provided as part of the manuscript or its supporting information, or deposited to a public repository. For example, in addition to summary statistics, the data points behind means, medians and variance measures should be available. If there are restrictions on publicly sharing data—e.g. participant privacy or use of data from a third party—those must be specified.

Reviewer #1: No

Reviewer #2: Yes

**********

5. Is the manuscript presented in an intelligible fashion and written in standard English?

PLOS ONE does not copyedit accepted manuscripts, so the language in submitted articles must be clear, correct, and unambiguous. Any typographical or grammatical errors should be corrected at revision, so please note any specific errors here.

Reviewer #1: Yes

Reviewer #2: Yes

**********

6. Review Comments to the Author

Please use the space provided to explain your answers to the questions above. You may also include additional comments for the author, including concerns about dual publication, research ethics, or publication ethics. (Please upload your review as an attachment if it exceeds 20,000 characters)

Reviewer #1: Thank you for asking me to review the revised manuscript which has improved and there is a more transparent methods section. I still have some concerns that are mainly focused on what the methodological framing of the study is – I do not find it to be a JLA study, rather a looser PSP. With some reframing in the manuscript, I think the study could be published as a PSP, but not as a JLA. The authors now note some of the divergent points in the manuscript and I have comments on some of these:

• There is a divergence between the PSP study in the manuscript and that of a JLA in generating potential research questions through surveys – these can be paper, online etc., and using focus groups additionally, and this is not fully reflected in the manuscript as a major methodological point.

• I am surprised to see the justification for not including informal carers as because they are rarely involved – this is contradicted by claims of the disabling nature of back pain and evidence on severe/high impact pain (and by my own clinical and research experience – may be a blind spot of this research team). At the minimum, a reference should be given here for that claim.

• The points about the recommended research priorities made by reviewer 2 also show divergence from a JLA approach. I am surprised that the authors haven’t taken this on more in their revisions.

I am not going to go into all of the divergent points, but this is why I think it is better to understand the study as a looser ‘PSP.’ I can see this research team have a similar study published as a JLA approach https://link.springer.com/article/10.1186/s41927-025-00588-7 and it is likely to be a contested point. I wonder if it would be best for the editor to contact the JLA directly with this query: https://www.jla.nihr.ac.uk/

Reviewer #2: The authors have addressed all of my previous comments satisfactorily. The revisions have improved the clarity, transparency, and interpretability of the manuscript, particularly with regard to methodological decisions and their underlying rationale.

The authors now clearly justify their choices, acknowledge potential limitations and sources of bias inherent to the methodology, and appropriately discuss the implications of these for interpretation of the findings. Overall, the manuscript presents a technically sound and well‑reported study, and I consider it suitable for publication.

**********

7. PLOS authors have the option to publish the peer review history of their article (what does this mean?). If published, this will include your full peer review and any attached files.

If you choose “no”, your identity will remain anonymous but your review may still be made public.

**Do you want your identity to be public for this peer review?**  For information about this choice, including consent withdrawal, please see our Privacy Policy.

Reviewer #1: No

Reviewer #2: No

**********

[NOTE: If reviewer comments were submitted as an attachment file, they will be attached to this email and accessible via the submission site. Please log into your account, locate the manuscript record, and check for the action link "View Attachments". If this link does not appear, there are no attachment files.]

To ensure your figures meet our technical requirements, please review our figure guidelines: https://journals.plos.org/plosone/s/figures

You may also use PLOS’s free figure tool, NAAS, to help you prepare publication quality figures: https://journals.plos.org/plosone/s/figures#loc-tools-for-figure-preparation.

NAAS will assess whether your figures meet our technical requirements by comparing each figure against our figure specifications.

Attachment Submitted filename: peer review PONE 17-5-26.docx

# Author comment: Author response to Decision Letter 2

**DOI:** 10.1371/journal.pone.0353164.r004

20 May 2026

Dear Editor and Reviewers,

Thank you for the time and effort you have invested into re-reviewing our article. We greatly appreciate your valuable comments and recommendations. We have addressed each of your comments in the point-by-point response below and a revised version of the manuscript has been submitted. Changes are highlighted in yellow in the “Revised Manuscript with Track Changes”. We believe these revisions have enhanced the quality of the manuscript, and we hope they meet your expectations, making it suitable for publication in PLOS One.

Sincerely,

Rikke Munk Killingmo, on behalf of all authors

Reviewer #1

Thank you for asking me to review the revised manuscript which has improved and there is a more transparent methods section. I still have some concerns that are mainly focused on what the methodological framing of the study is – I do not find it to be a JLA study, rather a looser PSP. With some reframing in the manuscript, I think the study could be published as a PSP, but not as a JLA. The authors now note some of the divergent points in the manuscript and I have comments on some of these:

Comment 1: There is a divergence between the PSP study in the manuscript and that of a JLA in generating potential research questions through surveys – these can be paper, online etc., and using focus groups additionally, and this is not fully reflected in the manuscript as a major methodological point.

Response:

Thank you for this comment. We agree that the use of focus groups instead of surveys represents an important methodological deviation from the traditional JLA PSP process.

In response to the previous review round, we added a dedicated section in the Materials and methods section describing how the study deviates from the JLA Guidebook. In this section, we clarify that uncertainties were identified through focus groups rather than surveys, and that this approach was chosen to ensure participant diversity and facilitate in-depth discussion and collective reflection: “…Fourth, uncertainties were identified through focus groups. While JLA PSPs typically use online surveys, focus groups and purposive recruitment were used to ensure participant diversity and enable in-depth discussion and collective reflection, revealing uncertainties that might not emerge through surveys.” The decision to adopt this approach was also informed by input from the Norwegian Spine and Back Pain Association regarding the method considered most appropriate to capture the perspectives of its members. This methodological choice is addressed as a potential limitation in the Discussion section.

To further address the reviewer’s concern, we have now explicitly emphasised in the manuscript that the use of focus groups rather than surveys constitutes a methodological adaptation of the traditional JLA PSP approach. Please refer to the Material and methods section or see below:

“Thus, the use of focus groups rather than surveys constituted a deliberate adaptation of the traditional JLA PSP approach.”

In the Discussion section, we further clarify that this decision was informed by input from the Norwegian Spine and Back Pain Association. Please refer to the Discussion section or see below:

“Thus, informed by input from the Norwegian Spine and Back Pain Association regarding the method considered most appropriate to capture the perspectives of its members, we chose focus group interviews ..”

Comment 2: I am surprised to see the justification for not including informal carers as because they are rarely involved – this is contradicted by claims of the disabling nature of back pain and evidence on severe/high impact pain (and by my own clinical and research experience – may be a blind spot of this research team). At the minimum, a reference should be given here for that claim.

Response:

Thank you for this important comment. We acknowledge that the original wording may have understated the role of informal caregiving in back pain management across different levels of severity.

We have therefore revised the sentence to better align with the broader literature on back pain. In particular, we have avoided implying that informal caregiving is absent and instead clarified that it is not typically a central component of routine management in the majority of cases. We have also added a supporting reference (Hartvigsen et al. 2018, What low back pain is and why we need to pay attention). Please refer to the Material and methods section or see below.

“Third, carers were not included as stakeholder group, as back pain is typically managed in primary care and largely self-managed, with informal caregiving not being a central component of routine management in most cases [2].”

Comment 3: The points about the recommended research priorities made by reviewer 2 also show divergence from a JLA approach. I am surprised that the authors haven’t taken this on more in their revisions. I am not going to go into all of the divergent points, but this is why I think it is better to understand the study as a looser ‘PSP.’ I can see this research team have a similar study published as a JLA approach https://link.springer.com/article/10.1186/s41927-025-00588-7 and it is likely to be a contested point. I wonder if it would be best for the editor to contact the JLA directly with this query: https://www.jla.nihr.ac.uk/

Response:

Thank you for this thoughtful comment. We appreciate the reviewer’s reflection on the degree of alignment between our study and the standard JLA PSP methodology.

We acknowledge that some aspects of our approach represent contextual adaptations of the JLA methodology, particularly regarding recruitment procedures and the use of focus groups for identifying uncertainties. However, these adaptations were undertaken in accordance with established JLA guidance, which allows methodological flexibility to ensure inclusivity and feasibility within specific contexts. Importantly, the core principles of the JLA PSP methodology were maintained throughout the study, including stakeholder involvement, transparent prioritisation of uncertainties, and a structured consensus-based process involving both patients and healthcare professionals.

Methodological adaptations within JLA PSPs are not uncommon. For example, previous JLA PSPs have reported adaptations while still explicitly positioning their studies within the JLA framework (e.g. Apea et al., 2025 https://doi.org/10.1136/bmjopen-2024-086922; Harris et al., 2022 https://doi.org/10.1136/bmjopen-2021-055780; Lyng et al., 2022 https://doi.org/10.1515/sjpain-2022-0019).

We have carefully re-reviewed the manuscript in light of this comment. We have clarified the methodological deviations and their rationale in the Methods section, as well as their implications in the Discussion section. We consider that the manuscript already addresses concerns regarding transparency of methodological alignment. However, to further improve clarity and address the reviewer’s concern regarding framing, we have revised the title of the manuscript to more explicitly reflect the adapted nature of our approach.

Please refer to the Title or see below:

“Research priorities for back pain: results from an adapted James Lind Alliance priority setting partnership”

We believe this revised wording more accurately reflects the study methodology and its contextual adaptations, while maintaining clear alignment with the JLA framework. We thank the reviewer for the opportunity to clarify and strengthen the transparency of our reporting.

Reviewer #2

The authors have addressed all of my previous comments satisfactorily. The revisions have improved the clarity, transparency, and interpretability of the manuscript, particularly with regard to methodological decisions and their underlying rationale. The authors now clearly justify their choices, acknowledge potential limitations and sources of bias inherent to the methodology, and appropriately discuss the implications of these for interpretation of the findings. Overall, the manuscript presents a technically sound and well reported study, and I consider it suitable for publication.

Attachment Submitted filename: Response to reviewer resubmisison JLA back pain.pdf

# Peer review: Decision Letter 2

**DOI:** 10.1371/journal.pone.0353164.r005

**Authors:** Alessandro Rodolico, Vincenzo De Luca, Vincenzo De Luca

21 Jun 2026

Research priorities for back pain: results from an adapted James Lind Alliance priority setting partnership

PONE-D-25-60540R2

Dear Dr. Killingmo,

We’re pleased to inform you that your manuscript has been judged scientifically suitable for publication and will be formally accepted for publication once it meets all outstanding technical requirements.

Within one week, you’ll receive an e-mail detailing the required amendments. When these have been addressed, you’ll receive a formal acceptance letter and your manuscript will be scheduled for publication.

An invoice will be generated when your article is formally accepted. Please note, if your institution has a publishing partnership with PLOS and your article meets the relevant criteria, all or part of your publication costs will be covered. Please make sure your user information is up-to-date by logging into Editorial Manager at Editorial Manager® and clicking the ‘Update My Information' link at the top of the page. For questions related to billing, please contact billing support.

If your institution or institutions have a press office, please notify them about your upcoming paper to help maximize its impact. If they’ll be preparing press materials, please inform our press team as soon as possible -- no later than 48 hours after receiving the formal acceptance. Your manuscript will remain under strict press embargo until 2 pm Eastern Time on the date of publication. For more information, please contact onepress@plos.org.

Kind regards,

Vincenzo De Luca

Academic Editor

PLOS One

Additional Editor Comments (optional):

Reviewers' comments:

Reviewer's Responses to Questions

**Comments to the Author**

1. If the authors have adequately addressed your comments raised in a previous round of review and you feel that this manuscript is now acceptable for publication, you may indicate that here to bypass the “Comments to the Author” section, enter your conflict of interest statement in the “Confidential to Editor” section, and submit your "Accept" recommendation.

Reviewer #1: All comments have been addressed

**********

2. Is the manuscript technically sound, and do the data support the conclusions?

The manuscript must describe a technically sound piece of scientific research with data that supports the conclusions. Experiments must have been conducted rigorously, with appropriate controls, replication, and sample sizes. The conclusions must be drawn appropriately based on the data presented.

Reviewer #1: Yes

**********

3. Has the statistical analysis been performed appropriately and rigorously?

Reviewer #1: N/A

**********

4. Have the authors made all data underlying the findings in their manuscript fully available?

The PLOS Data policy requires authors to make all data underlying the findings described in their manuscript fully available without restriction, with rare exception (please refer to the Data Availability Statement in the manuscript PDF file). The data should be provided as part of the manuscript or its supporting information, or deposited to a public repository. For example, in addition to summary statistics, the data points behind means, medians and variance measures should be available. If there are restrictions on publicly sharing data—e.g. participant privacy or use of data from a third party—those must be specified.

Reviewer #1: Yes

**********

5. Is the manuscript presented in an intelligible fashion and written in standard English?

PLOS ONE does not copyedit accepted manuscripts, so the language in submitted articles must be clear, correct, and unambiguous. Any typographical or grammatical errors should be corrected at revision, so please note any specific errors here.

Reviewer #1: Yes

**********

6. Review Comments to the Author

Please use the space provided to explain your answers to the questions above. You may also include additional comments for the author, including concerns about dual publication, research ethics, or publication ethics. (Please upload your review as an attachment if it exceeds 20,000 characters)

Reviewer #1: I contacted the JLA for advice regarding the phrasing used in studies that reported an adapted form of methods like your study and they suggested the following...We would typically see those referred to in publications using phrases like 'a priority setting project inspired by JLA methodology', or 'a priority setting project using adapated JLA methods'.

I have chosen 'accept' here for the manuscript as I don't think this warrants further review and you have addressed the issues raised in the reviews, but do think you should go through the abstract and manuscript and ensure consistency with the phrasing of the methodology to 'adapted JLA.'

**********

7. PLOS authors have the option to publish the peer review history of their article (what does this mean?). If published, this will include your full peer review and any attached files.

If you choose “no”, your identity will remain anonymous but your review may still be made public.

**Do you want your identity to be public for this peer review?**  For information about this choice, including consent withdrawal, please see our Privacy Policy.

Reviewer #1: No

**********

# Editor report: Acceptance letter

**DOI:** 10.1371/journal.pone.0353164.r006

**Authors:** Alessandro Rodolico, Vincenzo De Luca, Vincenzo De Luca

PONE-D-25-60540R2

PLOS One

Dear Dr. Killingmo,

I'm pleased to inform you that your manuscript has been deemed suitable for publication in PLOS One. Congratulations! Your manuscript is now being handed over to our production team.

At this stage, our production department will prepare your paper for publication. This includes ensuring the following:

* All references, tables, and figures are properly cited

* All relevant supporting information is included in the manuscript submission,

* There are no issues that prevent the paper from being properly typeset

You will receive further instructions from the production team, including instructions on how to review your proof when it is ready. Please keep in mind that we are working through a large volume of accepted articles, so please give us a few days to review your paper and let you know the next and final steps.

Lastly, if your institution or institutions have a press office, please let them know about your upcoming paper now to help maximize its impact. If they'll be preparing press materials, please inform our press team within the next 48 hours. Your manuscript will remain under strict press embargo until 2 pm Eastern Time on the date of publication. For more information, please contact onepress@plos.org.

You will receive an invoice from PLOS for your publication fee after your manuscript has reached the completed accept phase. If you receive an email requesting payment before acceptance or for any other service, this may be a phishing scheme. Learn how to identify phishing emails and protect your accounts at https://explore.plos.org/phishing.

If we can help with anything else, please email us at customercare@plos.org.

Thank you for submitting your work to PLOS ONE and supporting open access.

Kind regards,

PLOS ONE Editorial Office Staff

on behalf of

Dr. Vincenzo De Luca

Academic Editor

PLOS One
